# Spore type-specific gene expression profiles underlying development and leaf infection processes of *Colletotrichum graminicola*

**DOI:** 10.1093/g3journal/jkag254

**Published:** 2026-09-09

**Authors:** Disha Rathi, Karsten Andresen, Rolf Daniel, Marco Alexandre Guerreiro, Matthias Kretschmer, James Kronstad, Minou Nowrousian, Stefanie Pöggeler, Anja Poehlein, Lars M Voll, Daniela Elisabeth Nordzieke

**Affiliations:** Institute of Microbiology and Genetics, Department of Genetics of Eukaryotic Microorganisms, University of Göttingen, GZMB, Göttingen 37077, Lower Saxony, Germany; Institute of Molecular Physiology, Department of Microbiology and Biotechnology, Johannes Gutenberg University, Mainz 55128, Rhineland-Palatinate, Germany; Institute of Microbiology and Genetics, Genomic and Applied Microbiology & Göttingen Genomics Laboratory, Georg-August University of Göttingen, Göttingen 37077, Lower Saxony, Germany; Botanical Institute, Environmental Genomics Group, Christian-Albrechts University of Kiel, Kiel 24118, Schleswig-Holstein, Germany; Max Planck Institute for Evolutionary Biology, Plön 24306, Schleswig-Holstein, Germany; The Michael Smith Laboratories, Department of Microbiology and Immunology, University of British Columbia, Vancouver, British Columbia V6T 1Z4, Canada; The Michael Smith Laboratories, Department of Microbiology and Immunology, University of British Columbia, Vancouver, British Columbia V6T 1Z4, Canada; Department of Molecular and Cellular Botany, Ruhr-Universität Bochum, Bochum 44870, North Rhine-Westphalia, Germany; Institute of Microbiology and Genetics, Department of Genetics of Eukaryotic Microorganisms, University of Göttingen, GZMB, Göttingen 37077, Lower Saxony, Germany; Institute of Microbiology and Genetics, Genomic and Applied Microbiology & Göttingen Genomics Laboratory, Georg-August University of Göttingen, Göttingen 37077, Lower Saxony, Germany; Department of Biology, Molecular Plant Physiology Group, Marburg University, Marburg 35043, Hesse, Germany; Center for Synthetic Microbiology (SYNMIKRO), Marburg University, Marburg 35043, Hesse, Germany; Institute of Microbiology and Genetics, Department of Genetics of Eukaryotic Microorganisms, University of Göttingen, GZMB, Göttingen 37077, Lower Saxony, Germany; Department of Agricultural Sciences, Institute of Microbial Genetics, Molecular Plant-Pathogen Interaction Lab, BOKU University Vienna, Tulln/Donau 3430, Lower Austria, Austria

**Keywords:** *Colletotrichum graminicola*, RNA-seq, nanopore, oval conidia, falcate conidia, corn anthracnose, effectors, CAZymes, nutrient transporters

## Abstract

*Colletotrichum graminicola* causes significant losses of the staple crop maize worldwide. The fungus produces two distinct asexual spore types, oval and falcate conidia, which show unique processes in development and plant interaction. Based on genome resequencing of our laboratory strain (CgM2/M1.001), we investigated the gene expression profiles of oval and falcate conidia during development and early leaf infection using RNA-seq. Our results reveal specific gene expression profiles between the two spore types, indicating fundamental differences in their developmental programs that reflect different modes of infection. We identified expression patterns discriminating both conidia types from mycelium and spore type-specific ones for genes encoding transcription factors, conserved fungal developmental genes, transporters, genes of secondary metabolite clusters, and pathogenicity-related functions, including effectors and carbohydrate-active enzymes (CAZymes). Our study shows that despite the identical genomic basis, oval and falcate conidia show unique transcriptomes across vegetative development and early plant interaction. Taking together, these results provide new insights into the molecular mechanisms determining the biology of *C. graminicola* and its interaction with the plant host.

## Introduction

*Colletotrichum* is a genus of phytopathogenic fungi within the *Glomerellaceae* family and poses a significant threat to global food security by causing devastating diseases in numerous crop species consumed by humans (Gomes et al. 2021). *Colletotrichum* species interact with a broad range of plant hosts, including cereals (maize, rice, and sorghum), fruits (bananas, mango, avocado, citrus, and strawberry), legumes (soybean and common bean), and vegetables, which makes it a major constraint in global food production (Baroncelli et al. 2017; Joshi 2018; Samarakoon et al. 2018; Damm et al. 2019; Gomes et al. 2021; Talhinhas and Baroncelli 2021; Boufleur et al. 2022; Gupta et al. 2022; Liu et al. 2022). Species of the *Colletotrichum* genus are grouped into 15 species complexes and exhibit a remarkable diversity in their infection strategies, ranging from biotrophs, necrotrophs, and hemibiotrophs to endophytes, allowing them to colonize and obtain nutrients from their hosts in various ways (Baroncelli et al. 2017; De Silva et al. 2017; Talhinhas and Baroncelli 2021). The genus has been ranked as the eighth most important group of plant-pathogenic fungi globally, reflecting its significant scientific and economic impact (Cannon et al. 2012; Dean et al. 2012).

One of the most important *Colletotrichum* species infecting cereals is *Colletotrichum graminicola*, which primarily infects maize (*Zea mays*) and causes substantial losses worldwide (Frey et al. 2011; Belisário et al. 2022). Maize is among the world's most extensively cultivated crops, playing an essential role in global food systems as a fundamental source of nutrition for humans and animals and serving as a key raw material in various industrial applications including bioenergy (Kandil et al. 2020; Revilla et al. 2022; Assaf et al. 2024). *C. graminicola* can infect all plant parts and can be found throughout the growing season, making it a significant threat to maize production (Becerra et al. 2023). The disease manifests in anthracnose leaf blight (ALB), anthracnose stalk rot (ASR), and systemic plant infections, whereas current analyses estimate that ASR is the most important form causing 10–20% annual loss in maize harvest worldwide (Belisário et al. 2022). For efficient plant infection, *C. graminicola* produces two distinct spore types, oval and falcate conidia (Bergstrom and Nicholson 1999; Nordzieke et al. 2019b; Rudolph et al. 2025b). In contrast to other asexual spore type pairs from plant-pathogenic fungi like *Magnaporthe oryzae*, oval and falcate conidia both contain the full set of organelles and are able to germinate under favorable conditions (Panaccione et al. 1989; Zhang et al. 2014). However, the conidiation process of both spore types differs significantly: falcate conidia develop from conidiophores in acervuli on infected leaves, whereas oval conidia are formed from hyphae in parenchyma cells in both leaf and stem lesions (Sukno et al. 2008; Nordzieke et al. 2019b; Belisário et al. 2022). Recent characterization of both conidia types revealed that they show significant differences in spore hydrophobicity, developmental processes and the adaptation to leaf or root infection (Chaky et al. 2001; Nordzieke et al. 2019b; Rudolph et al. 2025b). Newly generated falcate conidia have a pronounced dormant stage, which is controlled by the secondary metabolites (SMs) mycosporine glutamine and mycosporine alanine (Leite 1992; Nordzieke et al. 2019b). Either exposure to glucose or attachment to hydrophobic surfaces on a polar adhesive strip induces a break of dormancy to give rise to either vegetative or pathogenic development, respectively (Leite 1992; Chaky et al. 2001; Vasselli et al. 2022). In contrast, oval conidia lack a dormant stage and start to germinate even under nutrient limited conditions (Nordzieke et al. 2019b). Oval conidia-derived germlings sense and grow toward each other by following chemical gradients to form a vegetative hyphal network by germling fusions, a process that has never been observed for falcate conidia so far (Nordzieke et al. 2019b; Rudolph et al. 2025a). The early developmental processes of both conidia types have consequences for the infection strategies on leaves: falcate conidia form melanized appressoria from short germ tubes which will subsequently form penetration pegs and infect the subjacent plant material. Oval conidia, however, form penetration structures from hyphae and germ tubes (hyphopodia) only when conidia are present in high spore densities. Thus, falcate conidia cause more severe symptoms on leaves when applied in lower spore numbers (Nordzieke et al. 2019b).

In the research community, the *C. graminicola* isolate CgM2 (M1.001), which was collected in Missouri from infected maize, is used for scientific investigations due to the reliable symptom development on leaves and stalks (Forgey et al. 1978). The first genome sequence of *C. graminicola* M1.001/CgM2 was generated in a community effort in 2012 using Sanger and 454 sequencing platforms, with a resulting reference assembly spanning 50.9 Mb. Due to the high number of repetitive sequences in the reference strains’ genome, the scaffolding was done based on the *Colletotrichum higginsianum* genome (O'Connell et al. 2012). A follow up study combined deep sequencing RNA-seq data with sophisticated bioinformatics strategies to improve genome annotation, which included identification of several hundred novel transcripts, improvement of gene models, and detection of candidate genes for alternative splicing (Schliebner et al. 2014). In 2023, a subsequent genome assembly of the same strain was generated using a combination of PacBio and Illumina sequencing technologies. Overall, 10 main chromosomes and 3 minichromosomes were identified, with a total genome size of 57.43 Mb, providing a high-quality genome sequence for this pathogen (Becerra et al. 2023). Those genomes were the bases of RNA-seq studies, focusing on different stages of leaf infection caused by falcate conidia. In those studies, genes encoding effectors, enzymes for SMs, transporters, hydrolases, and plant defense responses were identified as relevant for leaf infection by this spore type (O'Connell et al. 2012; Torres et al. 2016; Miranda et al. 2017).

In this study, we investigated gene expression profiles of oval and falcate conidia during development and early leaf infection via RNA-seq, aiming to explain the determining factors shaping spore type-specific behavior shown in previous work. To generate a sound basis for RNA-seq evaluation, we applied Nanopore and Illumina techniques to resequence the genome of our laboratory strain, which is present today in several lines around the globe, all originating from CgM2 (M1.001). We focused on the analysis of several functional gene groups relevant for development and pathogenicity, namely conserved fungal developmental genes and genes encoding for transcription factors (TFs), membrane transport proteins, secondary metabolite enzymes (encoded in gene clusters), effectors, and carbohydrate-active enzymes (CAZymes). Overall, we found significant overlaps and differences in gene expression profiles comparing oval conidia, falcate conidia and mycelium, indicating biological similarities and differences of the freshly harvested spores. Furthermore, we identified genes that encode pathogenicity relevant functions such as effectors, CAZymes, and transporters; some of these functions show spore type-specific expression patterns. The reported results support the view that oval and falcate conidia-derived hyphae have a specific identity which is maintained during development and early pathogenic interaction with the plant, shaping those processes in a spore type-specific way.

## Materials and methods

### Strains, media and growth conditions

The wildtype strain CgM2 (M1.001) of *C. graminicola* (Ces.) G.W.Wilson was provided by R. L. Nicholson to H. Deising (Martin-Luther University Halle-Wittenberg), who provided it to our laboratory. CgM2 was renamed to M1.001 by B. Hanau. Currently, both names are used as synonyms for the same strain (Forgey et al. 1978; Werner et al. 2007; O'Connell et al. 2012; Aliyeva-Schnorr et al. 2025). Falcate conidia were generated by growing *C. graminicola* on oatmeal agar (OMA, 1l: 50 g ground oat flakes (Alnatura, Darmstadt, Germany)) for 14–21 d. To stimulate falcate conidia generation, *C. graminicola* was exposed to red (660 to 665 nm) and blue (450 to 455 nm) light in a ratio of 3:1 at 23 °C. For the generation of oval conidia, mycelial plugs or a falcate conidia spore solution were inoculated in liquid complete medium with 0.5 M of sucrose (CMS, 1l: 55.5 mM glucose, 0.1% yeast extract, 0.1% peptone, 0.5 M sucrose, 5.3 mM Ca(NO_3_)_2_, 0.073 mM KH_2_PO_4_, 1.04 mM MgSO_4_, 0.46 mM NaCl) and shaken (80 g, 23 °C) in darkness for two days. After shaking, the cultivation was continued for 3 to 5 d in darkness without agitation (23 °C; Rudolph et al. 2025b). Mycelium was grown in liquid CM medium (CM, 1l: 1% glucose, 0.1% yeast extract, 0.1% peptone, 5.3 mM Ca(NO_3_)_2_, 0.073 mM KH_2_PO_4_, 1.04 mM MgSO_4_, 0.46 mM NaCl) using either mycelial plugs or spore solutions of the CgM2 wildtype strain, followed by cultivation for 5 d at 23 °C.

### Preparation of genomic DNA for genome resequencing

Genomic DNA (gDNA) was prepared from mycelium grown for 5 d in liquid CM medium. To allow the sequencing of full chromosomes of CgM2, the NucleoBond HMW DNA kit (Macherey-Nagel, Düren, Germany; ref 740160.2) was used for high molecular weight gDNA extraction according to the manufacturer's instructions.

Concentration of the isolated DNA was determined using the Qubit dsDNA HS Assay Kit as recommended by the manufacturer (Life Technologies GmbH, Darmstadt, Germany). Shotgun libraries were prepared using the Illumina DNA Prep, (M) Tagmentation kit and Nextera DNA CD Indexe kit (96 Indexes) as recommended by the manufacturer (Illumina Inc., San Diego, CA, USA). To assess quality and size of the libraries, samples were run on an Agilent Bioanalyzer 2100 using an Agilent High Sensitivity DNA Kit as recommended by the manufacturer (Agilent Technologies, Waldbronn, Germany). Concentrations of the libraries were determined using the Qubit dsDNA HS Assay Kit as recommended by the manufacturer (Life Technologies GmbH, Darmstadt, Germany). Sequencing was performed by using the NovaSeq6000 instrument (Illumina Inc., San Diego, CA, USA) using the NovaSeq6000 SP Reagent Kit (v1.5) and the NovaSeq XP 2-Lane Kit (v1.5) for sequencing in the paired-end mode 2 × 250 cycles.

For Nanopore sequencing 1.2 µg of high molecular weight DNA (HWD) was used for library preparation using the Ligation Sequencing Kit 1D (SQK-LSK109) as recommended by the manufacturer. Sequencing was performed for 72 h using a MinION device Mk1B and a SpotON Flow Cell R9.4.1 as recommended by the manufacturer (Oxford Nanopore Technologies) using MinKNOW software (22.05.5) for sequencing and Guppy (v7.1.3) in high accuracy mode for basecalling.

### Preparation of RNA samples and RNA extraction procedures

RNA of mycelium and freshly harvested oval and falcate conidia of wildtype CgM2 were isolated and prepared for subsequent sequencing. CgM2 falcate conidia were harvested after 28 d cultivation on OMA using 0.02% Tween 20 solution. Oval conidia were generated in liquid CMS medium and separated from mycelium by filtration (Miracloth, EMD Milipore Corp, Burlington, USA; ref 475855-1R). Both spore types were centrifuged (4,000 g, 10 min), the supernatant discarded and the pellet collected on a sterile filter disc (grade 3hw, Sartorius, Göttingen, Germany; LOT 10-038) with the help of a vacuum pump (Type: PM12640-026.3, Nr. 02128309, Biometra, Germany).

To assess gene expression during spore germination and germling fusion, distinct harvesting time points were chosen to reflect differences in conidia-specific developments. Oval conidia rapidly germinate also without the requirement of nutrient sources and reaches levels of more than 80% germination after 6 h. Germling network formation by oval conidia starts at that timepoint but reaches its maximum after about 17 h post inoculation (Nordzieke et al. 2019b). Germination of falcate conidia, however, is tightly controlled and several factors ensure spore dormancy und nutrient-limiting conditions (Leite and Nicholson 1992; Chaky et al. 2001; Vasselli et al. 2022). To identify genes responsible for the different progression of vegetative spore development, we first adapted the cultivation conditions to induce the desired developmental processes and allowed at the same time for germling harvest and RNA extraction. We found that inoculation of 50 µl of conidia diluted in 450 µl of distilled water together with 500 µl 50 mM NaNO_3_ solution (= 1 ml total volume) in 6 well plates (TC-Platte 6 Well Cell+,F; Sarstedt, Germany, ref 83.3920.300), which additionally contained pieces of miracloth (Miracloth, EMD Milipore Corp, Burlington, USA; ref 475855-1R) provided the required conditions to mimic development as observed previously on water agar (Nordzieke et al. 2019b). To ensure further oval conidia germination and germling fusion rates or dormancy of falcate conidia as observed previously, spore titers were adjusted for both spore types (falcate conidia (c = 2.1 × 10^7^ml^−1^) or oval conidia (c = 2.6 × 10^7^ml^−1^)). After incubation at 23 °C, liquid and fungal tissue were sampled by pipetting on a filter disc (grade 3hw, Sartorius, Göttingen, Germany; LOT 10-038) with the help of a vacuum pump (Type: PM12640-026.3, Nr. 02128309, Biometra, Germany). The dried filter was frozen in liquid nitrogen until RNA was extracted.

In a previous study, we found that oval and falcate conidia show a different behavior prior to leaf infection (Nordzieke et al. 2019b). Falcate conidia form appressoria from short germ tubes, followed by penetration of the plant surface. Oval conidia form networks by germling fusions when present in high spore densities. At the same time, hyphopodia, melanized penetration structures from hyphae, emerge and penetrate the plant surface (Nordzieke et al. 2019b). Although germling fusion and hyphopodia formation are spore concentration-dependent processes, network formation is not a prerequisite for the development of hyphopodia (Nordzieke 2022). To rule out this spore-density dependent factor when comparing transcriptomes of falcate and oval conidia early leaf infections, we choose spore titers enabling the generation of likewise symptoms strengths in both conidia types (Nordzieke et al. 2019b). To assess which genes are regulating the spore type-specific leaf infection strategies, we prepared RNA from infected maize leaves (cultivar Mikado, KWS SAAT SE, Einbeck, Germany). *Zea mays* plants were grown in a 4:1 mix of soil (SP Vermehrung, Einheiterde Werkverband e.V., Sinntal-Altengronau, Germany, Artikel-Nr. 11-01500-40) and vermiculite (grain size 2 to 3 mm, Isola Vermiculite GmbH, Sprockhövel, Germany) in a controlled environment (PK 520 WLED plant chamber (Poly Klima Climatic Growth System, Freising, Germany), day/night cycle of 12 h (26 °C/18 °C)) for 16 d. Secondary leaves of the grown plants were placed in square petri dishes (Petri Dish 100 × 100 × 20 mm, Sarstedt, 16 Park Way, Mawson Lakes, South Australia 5095, REF 82.9923.422) on top of wet blotting paper (BF2 580 × 600 mm, Sartorius, Göttingen Germany). Conidia suspensions were prepared in 0.01% Tween solutions to ensure attachment to the leaf surface (Gu et al. 2024). Then 10–12 drops, each of 10 µl c = 10^5^ ml^−1^), were applied to the leaves. The square plates were sealed to ensure high humidity. After incubation for 24 h at 23 °C, infection spots were collected (4-mm Miltex Biopsy Punch with Plunger, Integra, Mansfield, USA; ref 33-34-P/25) in an Eppendorf tube and stored in liquid nitrogen until RNA extraction.

RNA extraction was performed using a RNeasy Plant mini kit (Qiagen, Hilden, Germany, ref 74904). The eluted RNA was treated with DNase (40 μg) using Turbo DNA free kit (Invitrogen, Vilnius, Lithuania, ref AM1907) to remove DNA contamination. Clean-up of the samples was performed using a RNeasy mini elute clean-up kit (Qiagen, Hilden, Germany, ref 74204). Depending on the follow up application, RNA was either prepared for RNA sequencing or transcribed into complementary DNA (cDNA) as template for quantitative real-time PCR (qRT-PCR) (Supplementary Fig. 1).

### Validation of RNA-Seq data by qRT-PCR analysis

For qPCR, cDNA samples were prepared using purified RNA samples and an iScript cDNA synthesis kit (BIO RAD, CAT- 1708891). qRT-PCR was performed using SsoAdvanced universal SYBR green supermix (BIO RAD, CAT- 1725271). For detection, a CFX Connect Real-Time PCR detection system (Bio-Rad Laboratories, Hercules, CA, USA) was used. The GAPDH gene was used as the internal reference (housekeeping gene). Relative expression levels of target genes were calculated using the ΔΔCt method (Livak and Schmittgen 2001), normalizing each sample to its corresponding GAPDH value. Fold change values (log2 fold change ≤ −1 or ≥ 1, a value of −1 means that a gene is two-fold down-regulated, whereas a value of 1 means that the gene is two-fold up-regulated in this comparison) were applied for comparative analysis. Heatmaps representing the expression profiles from both RNA-seq and qRT-PCR Datasets were generated using the iDEP.96 platform (Ge et al. 2018).

### Genome assembly and annotation

Oxford Nanopore reads of at least 10 kb were assembled with Canu v2.2 (Koren et al. 2017) with parameter genome size = 58 m. The resulting assembly was corrected with four rounds of Racon v1.4.3 (Vaser et al. 2017) using the Oxford Nanopore reads and four rounds of Pilon v1.24 (Walker et al. 2014) using the trimmed Illumina reads. The resulting 17 contigs were compared with the previously published genome V1 including the published optical map data (O'Connell et al. 2012) to assign the contigs to chromosomes resulting in 13 contigs representing chromosomes. Racon and Pilon with the Nanopore and Illumina reads, respectively, were used to close the gaps introduced in the scaffolding. The resulting gapless contigs were analyzed for the presence of telomeric repeats (sequence TTAGGG) using a custom-made Perl script. Telomeric repeats were found at both ends of all contigs except for contig 10 that has a telomeric repeat at one end and 24 copies of the ribosomal RNA (rRNA) genes at the other end (most likely preventing assembly of telomeric repeats at that end). BUSCO (v.5.2.2) analysis (Manni et al. 2021) with BUSCO Dataset fungi odb10 showed 97.8% completeness.

For gene annotation, RNA-seq reads derived from the combined RNA of vegetative mycelium and two different spore types were assembled with Trinity v2.15.1 (Grabherr et al. 2011). The resulting *de novo* assembled transcripts were used together with the predicted proteins from the published V1 genome (O'Connell et al. 2012) for genome annotation with MAKER (v3.01.03; Cantarel et al. 2008) as well as for the BRAKER2 pipeline (v2.1.2; Stanke et al. 2006, 2008; Li et al. 2009; Hoff et al. 2016, 2019; Brůna et al. 2021). For the latter, two separate runs were conducted with protein and transcriptome input, respectively, and the resulting output files were combined using TSEBRA. The gene models from MAKER and BRAKER2 were combined using custom-made Perl scripts. Subsequently, another MAKER run was conducted using the predicted proteins and RNAs from the combined MAKER and BRAKER2 analyses together with predicted proteins from a recently published *C. graminicola* genome assembly (Becerra et al. 2023). BUSCO (Benchmarking Universal Single-Copy Orthologs) v.5.2.2 (Manni et al. 2021), analysis of the resulting predicted proteins with the fungi_odb10 Dataset showed 97.1% completeness.

### Annotation of relevant gene groups

#### TF-encoding genes

Data from the 312 predicted TF genes identified previously in *Neurospora crassa* OR74A (Carrillo et al. 2017) was used in this study. The predicted genome of *C. graminicola* was aligned with these predicted TFs using local BLAST+ command line homology searches. The matches of the discontiguous MegaBLAST were further scrutinized by a BLASTN (BLAST+ 2.15.0 version) search run against the predicted genome in order to remove TF-unrelated as well as mispredicted sequences. The final list contains all the TFs with e-value of <1e^−05^.

#### Effector encoding genes

Locus Tags of effector encoding genes were assigned based on previous effector gene identification performed by Becerra and coworkers (Becerra et al. 2023).

#### Genes encoding membrane transporters

Genes encoding membrane transport proteins were identified by a multilayered approach. Genes that had already been annotated in the genome version v4.0 (O'Connell et al. 2012) were identified by BLAST searches. The remaining genes were filtered by WoLF PSORT (https://wolfpsort.hgc.jp/) for plasma membrane, ER, vacuolar or mitochondrial localization and after that, by PFAM domain predictions typical for transport proteins (e.g. MFS_1, Sugar_tr, AA_permease, etc.). In this way, 104 novel transporter genes were identified. These candidate genes were then annotated by BLAST (version 2.14.0) searches against Transporter Classification Database (www.tcdb.org), the *Saccharomyces* Genome Database (www.yeastgenome.org) and the NCBI Reference Sequences database (www.ncbi.nlm.nih.gov), as described by (O'Connell et al. 2012).

#### Genes encoding CAZymes

The predicted proteome of *C. graminicola* was scanned against the dbCAN3 v11 database (Zheng et al. 2023) for CAZymes by using all tools (HMM, DIAMOND, dbCAN-sub) within the run_dbcan4 tool. Matches with an HMM e-value below 1e^−15^ and coverage greater than 0.35 were considered for further steps. Additionally, the proteome was screened by the CUPP online server (Barrett et al. 2020b) with default settings and considering matches with a significance score above 5. Matches identified exclusively by DIAMOND were excluded from further analyses. Protein classification was based on consistent matches across all used tools, while inconsistent matches were disregarded for subsequent CAZyme analyses. Substrate prediction was achieved by using the dbCAN_sub database (Zheng et al. 2023).

#### Genes encoding regulators of development

For identification of autophagy related proteins, proteins involved in signaling (MAP kinase pathways, NOX proteins, STRIPAK components, pheromone-signaling proteins), TFs involved in developmental processes and melanin biosynthesis proteins, a reciprocal BLASTP (version 2.0.10) and a BLASTN (BLAST+ 2.15.0 version) analysis was conducted based on *Sordaria macrospora* and *Aspergillus flavus* proteins sequences, respectively. The highest sequence identity and e-value threshold <1e^−05^ and a continuous overlap of 50% over the query sequence was used for the detection of *C. graminicola* homologs.

#### Secondary metabolite biosynthesis genes

In order to locate secondary metabolite biosynthesis gene clusters, the genome was analyzed with antiSMASH version fungiSMASH 7.0.0 (Blin et al. 2021).

#### RNA-seq analysis

RNA extraction was performed using a RNeasy Plant mini kit (Qiagen, Hilden, Germany, ref 74904). The eluted RNA was treated with DNase (40 μg) using Turbo DNA free kit (Invitrogen, Vilnius, Lithuania, ref AM1907) to remove DNA contamination. RNA was prepared for RNA sequencing by clean-up of the samples using a RNeasy mini elute clean-up kit (Qiagen, Hilden, Germany, ref 74204).

The NEB Next Poly(A) mRNA Magnetic Isolation Module (New England BioLabs, Frankfurt am Main, Germany) was used to reduce the amount of rRNA-derived sequences. For sequencing, the strand-specific cDNA libraries were constructed with a NEB Next Ultra II Directional RNA library preparation kit for Illumina and the NEB Next Multiplex Oligos for Illumina (96) (New England BioLabs, Frankfurt am Main, Germany). To assess quality and size of the libraries samples were run on an Agilent Bioanalyzer 2100 using an Agilent High Sensitivity DNA Kit as recommended by the manufacturer (Agilent Technologies). Concentration of the libraries was determined using the Qubit dsDNA HS Assay Kit as recommended by the manufacturer (Life Technologies GmbH, Darmstadt, Germany). Sequencing was performed on the NovaSeq 6000 instrument (Illumina Inc., San Diego, CA, USA) using NovaSeq 6000 SP Reagent Kit (100 cycles) and the NovaSeq XP 2-Lane Kit v1.5 for sequencing in the paired-end mode and running 2x 61 cycles.

RNA from 9 different samples with biologically independent triplicates per sample was analyzed by paired-end Illumina sequencing (Supplementary Table 1). Reads were mapped to the genome sequence with Hisat2 v2.2.1 (Kim et al. 2019) allowing multi-mapping with default parameters and parameters for intron lengths set to –min-intronlen 20 and –max-intronlen 4000. Reads mapping to annotated features were counted as described by (Teichert et al. 2012) with the modification that reads were strand-specific and counted if they mapped to the corresponding strand of the feature. Quantitative analysis of gene expression was done in R (v4.1.2; Team 2024) with DESeq2 1.34.0 (Love et al. 2014) using independent filtering in the results function. Genes were identified as differentially expressed if they had an adjusted *P*-value (Padj) of <0.05 and log2 values of fold expression changes were either ≥ 1 for up-regulated or ≤−1 for down-regulated genes. Venn diagrams of genes that were up- or down-regulated in different combinations were generated by identifying the corresponding genes using custom-made perl scripts (File S2), downstream processing using sorting functions in Excel (Microsoft) and generating of the diagram in Adobe Illustrator. The top 100 variable genes were identified using iDEP.96 (Ge et al. 2018) using the following criteria: log2 fold change ≤ −1 or ≥1, padj <0.05. In iDEP.96, genes are ranked by standard deviation across all samples. The top 100 genes are used in hierarchical clustering using the heatmap.2 function. In the generated heatmaps, the data is centered by subtracting the average expression level for each gene. The distance matrix is 1- r, where r is Pearson's correlation coefficient. The average linkage is used. The sample groups are not used in hierarchical clustering; they are just shown as color bars. The correlation matrix is computed using the cor function in R and does not use the bottom 25% of genes regarding expression level. The final graph is generated using ggplot2 (Ge et al. 2018). The generation of volcano blots was performed using the database VolcaNoseR (https://huygens.science.uva.nl/VolcaNoseR/; Goedhart and Luijsterburg 2020). VolcaNoseR settings were as follows: fold change threshold: −2 to 2; significant threshold: 2; use thresholds to annotate: changed and significant; criterion for ranking hits: Manhattan distance. Pathway enrichment analysis was performed using the GAGE method implemented in iDEP.96 (log2 fold change ≤−1 or ≥1, padj <0.05). Gene sets ranging from 5 to 2,000 genes were included. Pathways were considered significant at an FDR cutoff of 0.05. Prior to enrichment analysis, genes with FDR ≥ 1 were excluded.

#### Application of AI tools

The manuscript text was edited to improve spelling and grammar using the AI tools “Chat AI” (version v0.9.0) of ,Gesellschaft für wissenschaftliche Datenverarbeitung mbH Göttingen‘, GWDG and “Academic AI’ of ACOnet, Austria.

## Results

### Genome resequencing of the *C. graminicola* laboratory strain CgM2 (M1.001)

Since its collection, the CgM2/M1.001 isolate is used in several labs around the world. We therefore performed resequencing of our laboratory strain in this study to generate a reliable basis for data evaluation and interpretation. In this genome version V5, we obtained a gapless assembly with telomeric repeats at both ends of most chromosomes apart from chromosome 10, which contains rDNA repeats (File S3-17). In total, we assembled 13 chromosomes for *C. graminicola*, including the minichromosomes Chr11, Chr12 and Chr13 (Fig. 1). All chromosomes sum up to 57,600,233 base pairs in total. Using RNA-seq of a mixed sample containing biological material of *C. graminicola* mycelium, oval, and falcate conidia, 15,481 protein-encoding genes were annotated (Dataset S1). Although chromosome 1 is the longest, spanning 7,645,418 base pairs with 2,013 predicted genes, chromosome 2 contains the highest number of predicted genes (2,289). In total, our genome version V5 is very similar to V4 (Becerra et al. 2023), however, the total number of encoded proteins in V5 is increased by 400 (Table 1), most likely because different input data and algorithms were used for annotation.

**Fig. 1.**
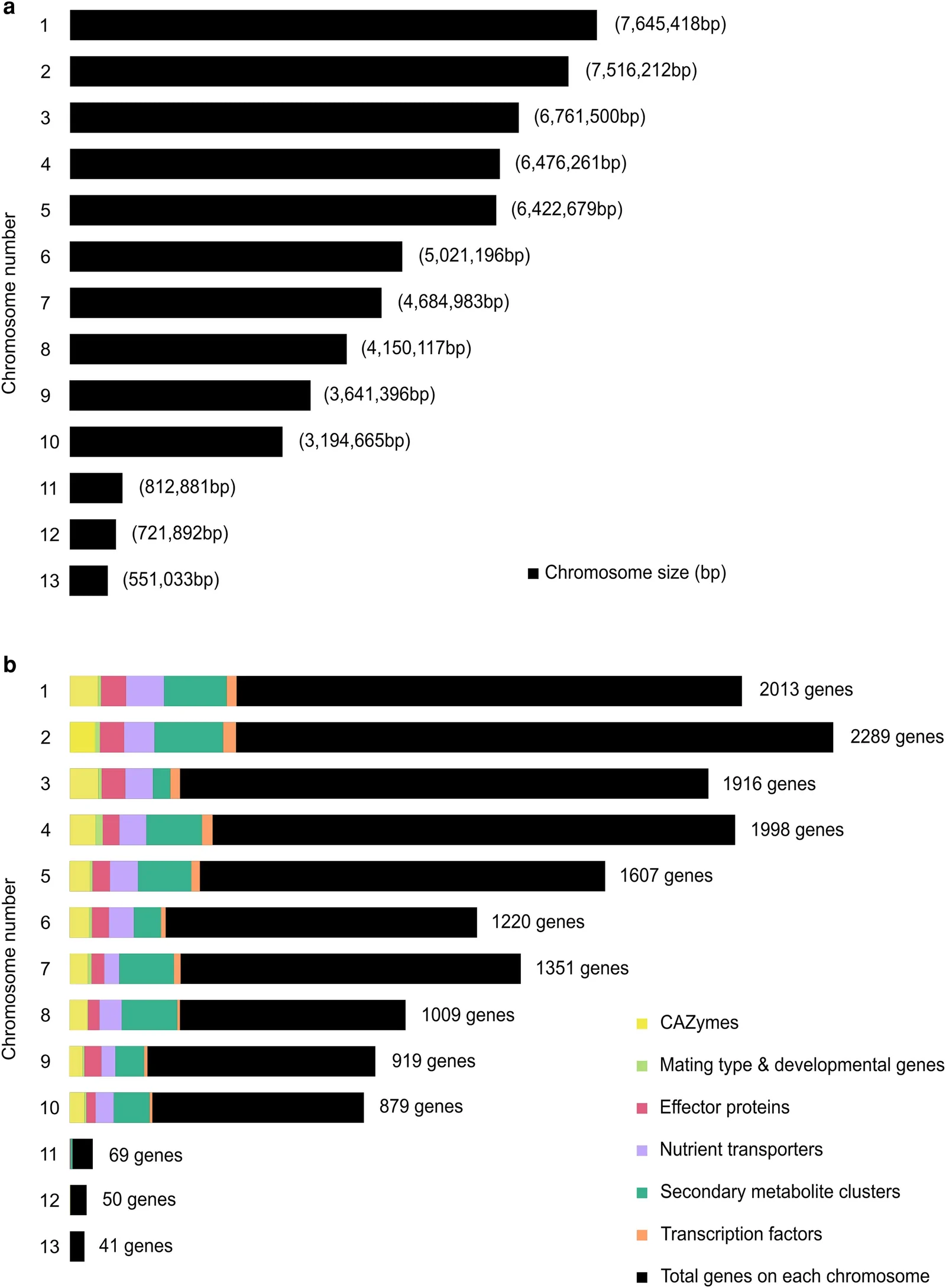
Overview of the *C. graminicola* assembly V5. a) Size of different chromosomes in base pairs (bp). b) Annotation of different functional gene groups based on the number of genes on 13 chromosomes, colored bars represent different functional categories. The black bar represents the total number of genes on one chromosome, including colored functionally categorized genes.

**Table 1.** Comparison of assembly statistics for the *C. graminicola* M1.001 genome.

Description	V1	V4	V5
No. of contigs	1,151	13	13
Size (Mb)	50.9	57.43	57.60
Number of protein coding genes	12,006	15,118	15,481
GC content (%)	49.12	45.98	46

V1 (O'Connell et al. 2012), V4 (Becerra et al. 2023), and V5, which was generated in this study applying a combination of Nanopore and Illumina sequencing techniques.

### Differential gene expression dynamics during spore development and maize leaf infection

In the 1970s, it was discovered that *C. graminicola* generates two distinct spore types (Nishihara 1975). Recent investigations from our group provided evidence that the differences culminate in specialization of the two spore types for the infection of different plant tissues (Nordzieke et al. 2019b; Rudolph et al. 2025b). In this study, we performed a comparative RNA-seq analysis to unravel the genetic basis for the observed biological differences between the spore types, focusing on three relevant timepoints identified in earlier work: cultivation under nutrient depletion conditions for 5 and 16 h induce different developmental programs. In oval conidia, germination is induced after 5 h, which is followed by the generation of germling fusion, an active process at 16 h. Falcate conidia, however, remain dormant during both time points (Nordzieke et al. 2019b). In addition to the vegetative development, we included early leaf infection (1 dpi) samples. At this stage, high percentages of falcate conidia have formed penetrating appressoria on short germ tubes. Oval conidia, on the other hand, did germinate on leaves followed by the formation of germling fusions and penetrating hyphopodia. Originating from appressoria and hyphopodia alike, plant invasion has started and biotrophic primary hyphae are visible *in planta* (Nordzieke et al. 2019b; Nordzieke 2022). As controls, we included samples of freshly harvested oval and falcate conidia as well as mycelium of *C. graminicola*.

A Principal Component Analysis (PCA) was conducted to assess the quality of the data generated, and to visualize sample clustering and variation among the biological replicates (Fig. 2a). In our study, PC1 explains 44% of the total variance, while PC2 accounts for an additional 20%, indicating that these two components together capture a substantial 64% of the transcriptomic variation. Biological replicates of the different samples clearly cluster according to the two spore types (falcate and oval conidia), and leaf infection (1 dpi) with mycelium as an outgroup. Notably, falcate conidia (Fc) and oval conidia (Oc) samples were separated by PC1, demonstrating strong transcriptional differences between the two spore types. Within each group, samples taken during early vegetative development (5 h, 16 h) separate clearly, suggesting stage-specific expression changes. Mycelium samples (Myc) cluster apart from all conidial stages, emphasizing a specific expression proﬁle during vegetative growth. The separation of leaf infection samples (Fc1dLF, Oc1dLF) from other samples predominantly by PC2 further supports the presence of transcriptional reprogramming during host-pathogen interaction.

**Fig. 2.**
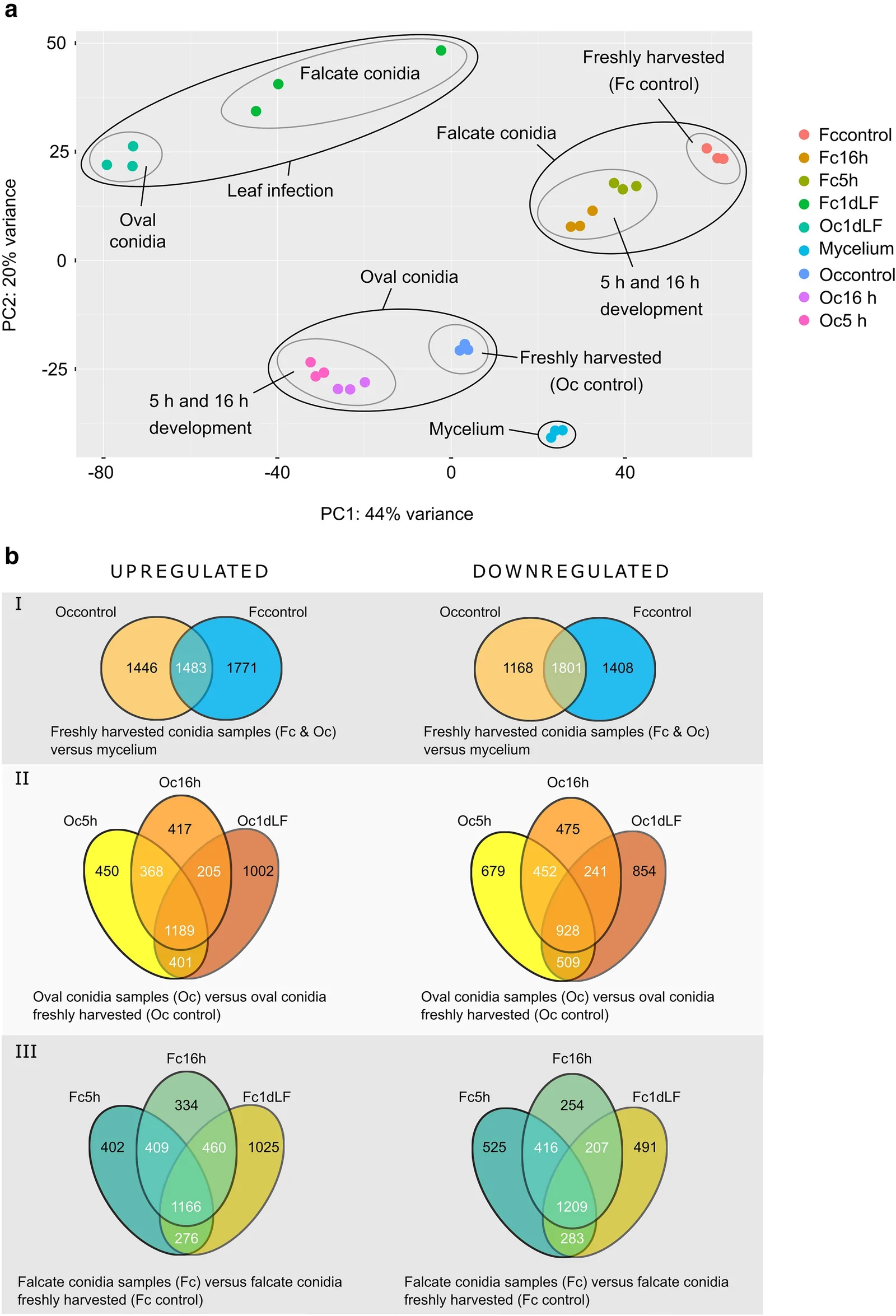
Differentially expressed genes in two different conidia types identified by DESeq2. Genes were identified as differentially expressed if they had an adjusted P-value (P_adj_) of <0.05 and log2 values of fold expression changes were either ≥ 1 for up-regulated or ≤ −1 for down-regulated genes. a) Principal component analysis of RNA-seq data for all samples. The first two principal components explain 64% of variance. The four major groups of samples consist of mycelial samples, oval conidia (w/o the infection samples), falcate conidia (w/o the infection samples), and leaf infection samples. Oval conidia are displayed to improve visibility, they do not have statistical significance. b) The Venn diagrams present an overview of differentially regulated genes of (I) freshly harvested falcate and oval conidia, (II, III) developmental stages of oval conidia (II) and falcate conidia (III).

Visualization of the globally up- and down-regulated genes using Venn diagrams was employed to characterize differential gene expression during spore development and leaf infection (Fig. 2b). Reference samples were chosen dependent on the biological sample analyzed. Freshly harvested oval and falcate conidia were compared with mycelial samples to determine whether these morphologically distinct stages differ in their transcriptomes. For all stages developing from freshly harvested conidia (5 h, 16 h, 1 d leaf infection), the corresponding freshly harvested conidia sample served as control. To reflect probable differences between early leaf infections of both conidia types, DEGs between infection samples of oval and falcate conidia were identified.

In freshly harvested oval and falcate conidia, >1,000 genes were differentially regulated relative to vegetative mycelium and shared among both spore types (1,483 up-regulated, 1,801 down-regulated), indicating that oval and falcate conidia are intrinsically diverse from vegetative hyphae (Fig. 2b). At the same time, >1,000 genes are regulated in a spore type-specific manner relative to vegetative mycelium (oval conidia: 1,446 up-regulated, 1,168 down-regulated; falcate conidia: 1,168 up-regulated, 1,408 down-regulated), underpinning the different nature of those conidia types compared to each other and reflecting the results obtained by PCA analysis (Fig. 2a). Furthermore, we observed differences in gene expression between the two conidia types during developmental transitions, 5 and 16 h inoculations under nutrient-limiting conditions, and early leaf infection (1 dpi). In falcate conidia, 665 genes were differentially up-regulated and 838 were differentially down-regulated across all developmental stages and during early leaf infection. Oval conidia displayed even stronger transcriptional dynamics, with 1,166 genes up-regulated and 929 down-regulated, reflecting a more pronounced transcriptional reprogramming associated with developmental progression and pathogenesis.

To obtain additional insight, we examined the 100 most variable expressed genes across developmental transitions, including freshly harvested conidia (control), critical timepoints for vegetative development (5 h, 16 h), and maize leaf infection (1 dpi, Supplementary Figs. 2-5). Pathway enrichment analysis performed with iDEP.96 revealed distinct expression signatures of freshly harvested oval and falcate conidia and early infection stages (Supplementary Table 2). Specifically, genes involved in rRNA processing, cellular responses to DNA damage, and general stress responses were predominantly up-regulated in freshly harvested conidia. In contrast, during early leaf infection (1 dpi), there was significant enrichment for pathways linked to secondary metabolite biosynthesis, carbohydrate metabolism, and hydrolase activity.

### Validation of differential gene expression by qRT-PCR

To confirm the transcriptomic data obtained from RNA-seq (Dataset S2), a subset of differentially expressed genes was validated using quantitative real-time PCR (qRT-PCR, Fig. 3, Supplementary Table 3). The selection was based on the significance of the RNA-seq data (top 20 up- and down-regulated genes) and interesting regulation patterns dependent on spore type, developmental stage or early infection processes. Among the selected seven genes, COGRA5_13220 (Emopamil binding protein), COGRA5_06545 (Cutinase), COGRA5_00514 (Apoplastic effector) and COGRA5_09579 (Fungal RiPP-like secondary metabolite) showed interesting expression patterns, indicating a probable role of these in specific developmental transitions, such as dormancy (COGRA5_13220), dormancy breaking of falcate conidia (COGRA5_06545), germination induction of oval conidia (COGRA5_00514), and adhesion (COGRA5_09579). Regarding early leaf infection, we selected COGRA5_00442 (Carboxylesterase), which is overexpressed in falcate conidia during maize leaf infection, while COGRA5_14555 (Taurine catabolism dioxygenase TauD) showed increased expression in oval conidia under the same condition (Dataset S3). In contrast, COGRA5_09579 (Fungal RiPP-like secondary metabolite) is up-regulated in both spore types during infection, indicating shared pathogenicity-related functions. Overall, expression proﬁles obtained from qRT-PCR (Supplementary Fig. 6) were like the RNA-seq results and thus confirmed the robustness of the RNA-seq findings.

**Fig. 3.**
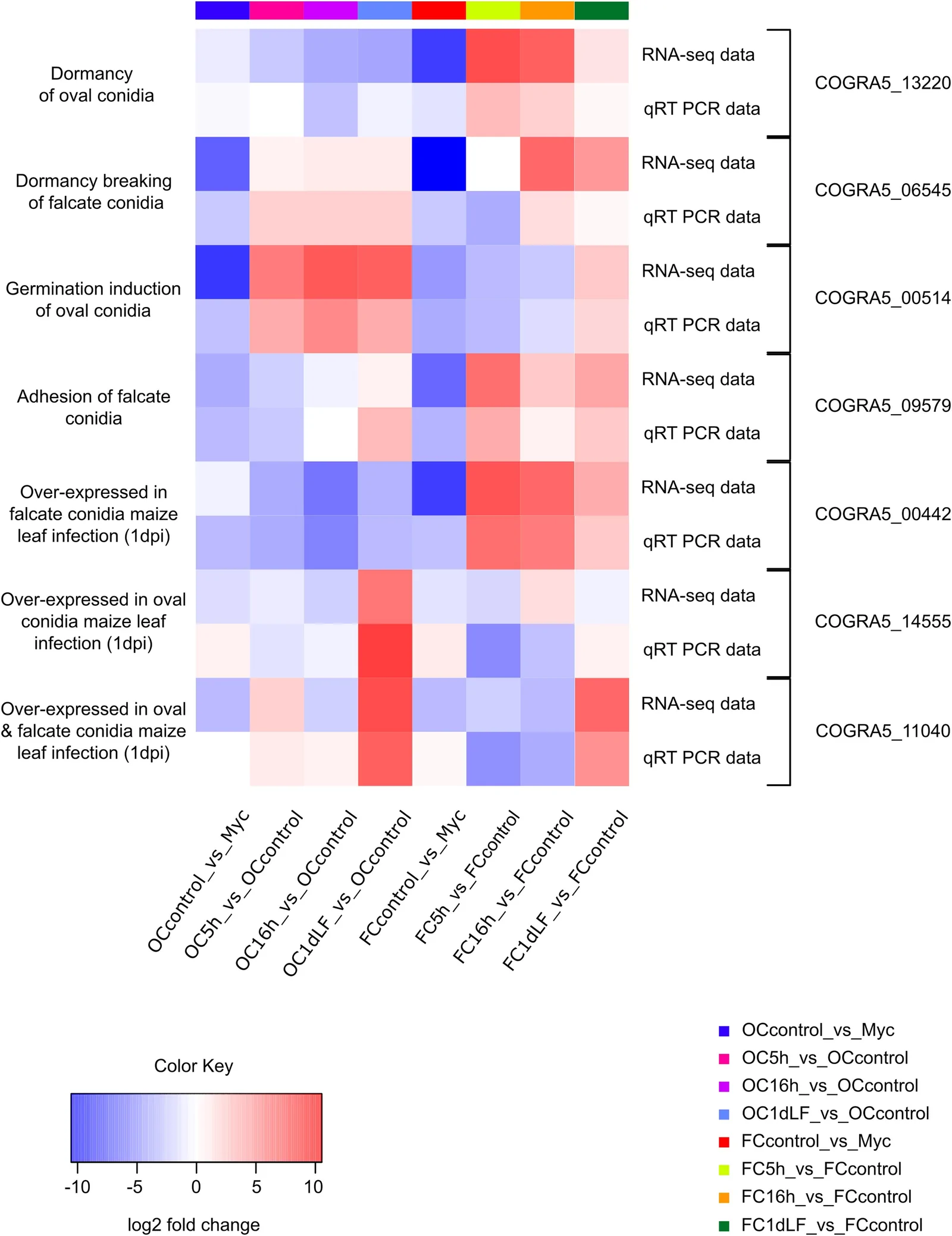
Comparative analysis of transcriptomic expression using both qRT-PCR and RNA-seq of selected genes. The data reflects the fold change correlations between these two methods, presented as a heat map for selected genes with a potential role in distinct developmental stages of falcate and oval conidia indicated (log2 fold change ≤−1 or ≥1, padj <0.05). The heatmap was created using iDEP.96 (Ge et al. 2018). Red color depicts the up-regulated genes, whereas blue represents the down-regulated genes.

### Annotation and transcriptome proﬁling of oval and falcate conidia across different functional gene groups

To gain an overview of the encoded genes, we performed a comprehensive annotation of all predicted genes with respect to their putative roles in fungal development (conserved regulators of development, TFs, SMs) and pathogenicity (CAZymes, effectors, nutrient transporters; Fig. 1b). All analyzed functional gene groups are broadly distributed across all ten major chromosomes (Chr 1–10). In contrast, the microchromosomes (MCs) in *C. graminicola* are enriched in repeats and have reduced gene content compared to the core chromosomes. The MCs harbor a limited number of annotated genes, with approximately 84% of the 160 genes being hypothetical. Interestingly, some of the annotated genes on the MCs are related to pathogenicity, including COGRA5_15372 (minichromosome 11), which encodes glutathionylspermidine synthase and has been previously shown to contribute to virulence in *C. graminicola* (Jaramillo et al. 2015). Our RNA-seq data analysis also revealed strong induction of COGRA5_15372 during early leaf infection (1 dpi) by falcate conidia, suggesting a role in plant penetration and establishment of infection. Furthermore, the minichromosome 12 and 13 harbor 4 ankyrin repeat proteins, which are versatile protein motifs involved in mediating diverse protein-protein interactions (Bork 1993; Mosavi et al. 2002). In fungi, ankyrin repeat proteins have been shown to function as TFs or regulators of secondary metabolite biosynthesis, including toxins that contribute to virulence (Pedley and Walton 2001).

#### TF-encoding genes

TFs are proteins that bind to specific DNA sequences and regulate gene expression. All eukaryotes, including fungi, depend on appropriately orchestrated TFs to coordinate the expression of genes essential for individual developmental stages and for the induction of vital metabolic pathways. These pathways encompass carbohydrate, iron, and nitrogen metabolism, as well as tolerance to oxidative stress, osmotic conditions, pH levels and UV light. Furthermore, TFs are instrumental in establishing developmental processes like vegetative growth, tissue differentiation (fruiting bodies, conidiophores), and pathogenicity (John et al. 2021).

Analysis of the *C. graminicola* genome revealed 216 identified TFs representing a diverse array of gene families (Dataset S4). The Zn_2_C_6_ (GAL4-like zinc cluster) and C_2_H_2_ zinc finger families were the most prevalent, consistent with their known abundance and regulatory significance in plant-pathogenic ascomycetes. Analysis of differentially expressed TF genes revealed that most were more highly expressed in freshly harvested conidia, with a general tendency toward down-regulation during subsequent development and maize leaf infection (Fig. 4a). The 100 most variable TF genes were identified using iDEP.96 as described in the Materials and Methods section. This analysis highlights genes with opposite regulatory patterns in oval and falcate conidia across the analyzed stages (Fig. 4b): in freshly harvested falcate conidia, specific TFs were down-regulated, whereas their expression increased during all developmental and pathogenicity stages. In contrast, oval conidia showed the opposite pattern, with up-regulation in freshly harvested spores and down-regulation in later stages. Notable examples include the DEGs COGRA5_02342 (ascospore maturation protein; KilA-N domain), COGRA5_07325 (Zn cluster TF; Zinc finger), and COGRA5_03862 (homeobox domain-containing protein; Homeobox domain). In contrast, for TFs up-regulated during oval conidia development, we did not find such clear differences to falcate conidia (Supplementary Fig. 7).

**Fig. 4.**
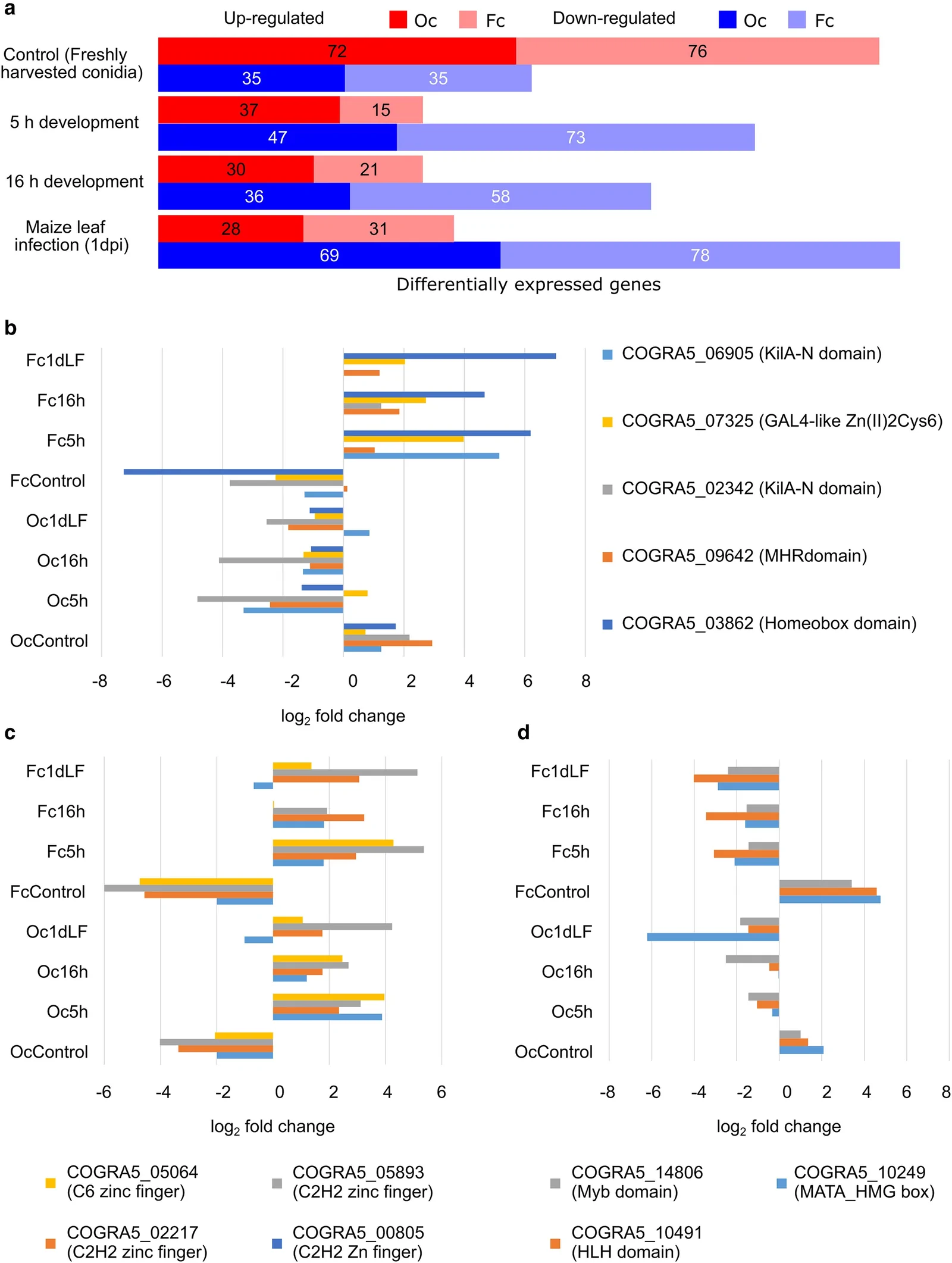
Expression patterns of transcription factor-encoding genes in oval and falcate conidia. Genes were identified using DESeq2 as differentially expressed if they had an adjusted P-value (P_adj_) of <0.05 and log2 values of fold expression changes were either ≥1 for up-regulated or ≤−1 for down-regulated genes. The mycelial sample served as the reference for freshly harvested conidia, whereas freshly harvested conidia were used as the reference for the 5 h, 16 h, and early leaf infection samples. a) Total number of predicted transcription factor genes showing differential regulation (log2 fold change ≤−1 or ≥1, padj <0.05). Red color depicts the up-regulated genes, whereas blue represents the down-regulated genes. (b-d) log2 fold change ≤−1 or ≥1, padj <0.05 and different color bar represent different transcription factor genes. The top 100 variable genes were identified using iDEP.96 (Ge et al. 2018). b) Transcription factor-encoding genes with induced expression for falcate conidia, as compared to oval conidia (c, d) Transcription factor genes (c) up-regulated and (d) down-regulated in both spore types during different developmental stages.

Additionally, several TFs exhibited similar regulation patterns in both conidial types across developmental stages and leaf infection (1 dpi), such as the DEGs COGRA5_05064 (transcriptional regulatory protein Pro1, C6 zinc finger) and COGRA5_05893 (nuclear division 74 protein; C2H2 zinc finger), were consistently up-regulated during development and leaf infection (Fig. 4c). Conversely, others including the DEGs COGRA5_14806 (Myb-like DNA binding protein; Myb domain) and COGRA5_10249 (female and male fertility protein; MATA_HMG box) were down-regulated in both conidia types (Fig. 4d).

#### Effector encoding genes

Fungal effectors are secreted proteins or small molecules that facilitate host colonization by modulating plant immune responses. These molecules are differentially expressed based on the contacting host tissue and stages of disease development in plant-pathogenic species (Sonah et al. 2016). Depending on their mode of action and subcellular localization, effectors can be broadly categorized as apoplastic (extracellular) or cytoplasmic (intracellular; Todd et al. 2022).

Apoplastic effectors act in the extracellular space of plant tissues, e.g. by binding to fungal cell wall components such as chitin to prevent host recognition, whereas cytoplasmic effectors are delivered inside plant cells, where they interfere with intracellular immune signaling and defense mechanisms (Li et al. 2024). Based on the 523 annotated effector encoding genes of genome version 4 (Becerra et al. 2023), 513 putative effector genes were identified, comprising 295 apoplastic, 159 cytoplasmic and 59 apoplastic\cytoplasmic candidates (Dataset S5). To get an indication, whether effector gene expression is regulated similarly in both spore types, the Top 100 most variable genes were identified using iDEP.96 as described in the material and methods section (Supplementary Fig. 8). A comparison of effector gene expression patterns within the top 100 genes revealed that most are similarly regulated during developmental and pathogenic processes in oval and falcate conidia, indicating that both spore types interact with the same environment and host. However, several effector genes show distinct and even opposite regulation in the 1 dpi infection stage. To analyze these initial findings in more detail, volcano plots were generated using VolcaNoseR on the basis of gene expression patterns of all effector genes identified (https://huygens.science.uva.nl/VolcaNoseR/; Supplementary Fig. 9). As expected, several effector genes were significantly induced during leaf infection as compared to freshly harvested conidia in both spore types. Interestingly, several of the top 10 significantly induced effector genes identified were shared among both spore types, including COGRA5_07713 (apoplastic effector), COGRA5_11054 (cytoplasmic effector), and COGRA5_03609 (apoplastic/cytoplasmic effector). However, when we used volcano plotting to visualize differences in between early leaf infection of both spore types, some effectors were visualized exhibiting opposing patterns of regulation (Fig. 5, Supplementary Table 4). For example, the known apoplastic effector COGRA5_04185 encoding a hydrophobin (Bettini et al. 2012; Eisermann et al. 2019) is strongly induced during leaf infection by falcate conidia but is not regulated in leaves infections of oval conidia. COGRA5_10559, previously described as a switch-specific effector (Kleemann et al. 2012; Oome and Van den Ackerveken 2014; Torres et al. 2016; Seidl and Van den Ackerveken 2019), was significantly up-regulated when comparing falcate and oval conidia infections.

**Fig. 5.**
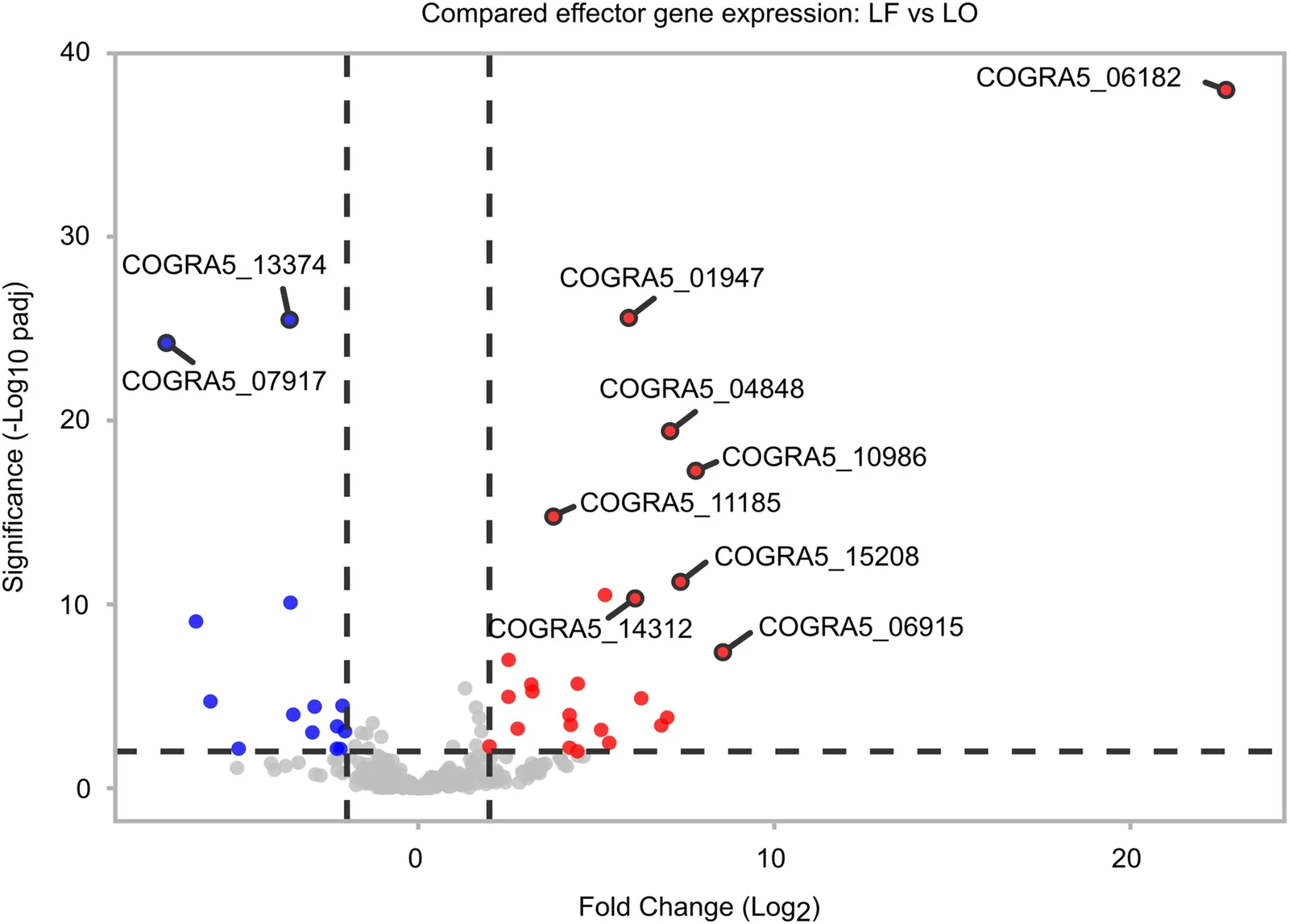
Volcano plot of effector genes during early leaf infection by oval and falcate conidia. The volcano plot was generated based on the comparison of gene expressions of all identified effector genes of falcate conidia leaf infection vs oval conidia leaf infection. VolcaNoseR settings were as follows: fold change threshold: −2 to 2; significant threshold: 2; use thresholds to annotate: changed and significant; criterion for ranking hits: Manhattan distance. Significantly regulated genes comparing falcate conidia leaf infection and oval conidia leaf infection are indicated in red (up-regulated) and blue (down-regulated). Locus tag numbers of the 10 most relevant hits are indicated.

#### Genes encoding membrane transporters

Transporters facilitate selective movement (import or export) of molecules across membranes. Organic and inorganic nutrients like sugars, amino acids, ions, or other metabolites, and water are likely to be taken up across the plasma membrane (Dahl et al. 2004). Transporters play a crucial role in securing survival, growth, morphogenesis, and pathogenesis by enabling the pathogen to adapt to different nutritional conditions, which are faced in and outside host cells (Verma et al. 2014; Lange and Peiter 2020). The differential expression of transporter-encoding genes can therefore indicate metabolic shifts in the host, transition of fungal developmental stages, and spore type-specific physiological strategies.

From the total of 738 predicted transporter-encoding genes in *C. graminicola*, including 104 newly annotated transporter genes (Dataset S6), we first analyzed the Top 100 genes showing the strongest variability in their expression, as identified by using iDEP.96 with criteria described in the material and methods section. The generated heatmap depicted three main regulatory groups, which each contain about a third of all genes included in this analysis (Supplementary Fig. 10). Two groups contained transporter-encoding genes which show a general opposite regulation pattern in oval and falcate conidia over all developmental and pathogenicity stages investigated. Gene regulations in the third group do not show variability among oval and falcate conidia, but a general induction during early stages of leaf infection compared to vegetative development. Thus, these transporters might be involved in the adaptation of nutrition acquisition from *ex* to *in planta* environment. When we analyzed the transporter-encoding genes having the highest expression during early leaf infection in more detail, we found that within the 40 highest expressed genes there were several transporter families showing similar induction in oval and falcate conidia (Fig. 6). As most prevalent, those include AAAP type amino acid permeases and sugar porters (SP). At the same time, several transporter families were over- or underrepresented when the two spore types were compared. Those include transporters from the DHA1 and DHA2 families, which are much more frequently induced in falcate than in oval conidia. Also in other respects, the profile of induced transporters differs strongly between oval and falcate conidia. 48% of the top 40 induced transporter-encoding genes in oval conidia were anion:cation symporters (ACS), whereas the same transporter family sums up to 20% in falcate conidia, indicating a probable spore type-specific strategy for the uptake of carboxylates such as succinate and citrate, organic acids and vitamins (Nie et al. 2017). We further identified several other transporter gene families that show clear spore type-specific expression patterns, such as monocarboxylate porter (MCP), p-ATPases, and tricarboxylate carriers, which are prominently up-regulated in falcate conidia, indicating adaptation to a nutrient environment rich in carboxylic acids (like C4 leaves). In oval conidia, several members from the oligopeptide transporter (OPT) and L-type amino acid transporter (LAT) families were induced. Since these were absent among induced transporter genes in falcate conidia, our results point to a preference of these nitrogen sources in oval conidia.

**Fig. 6.**
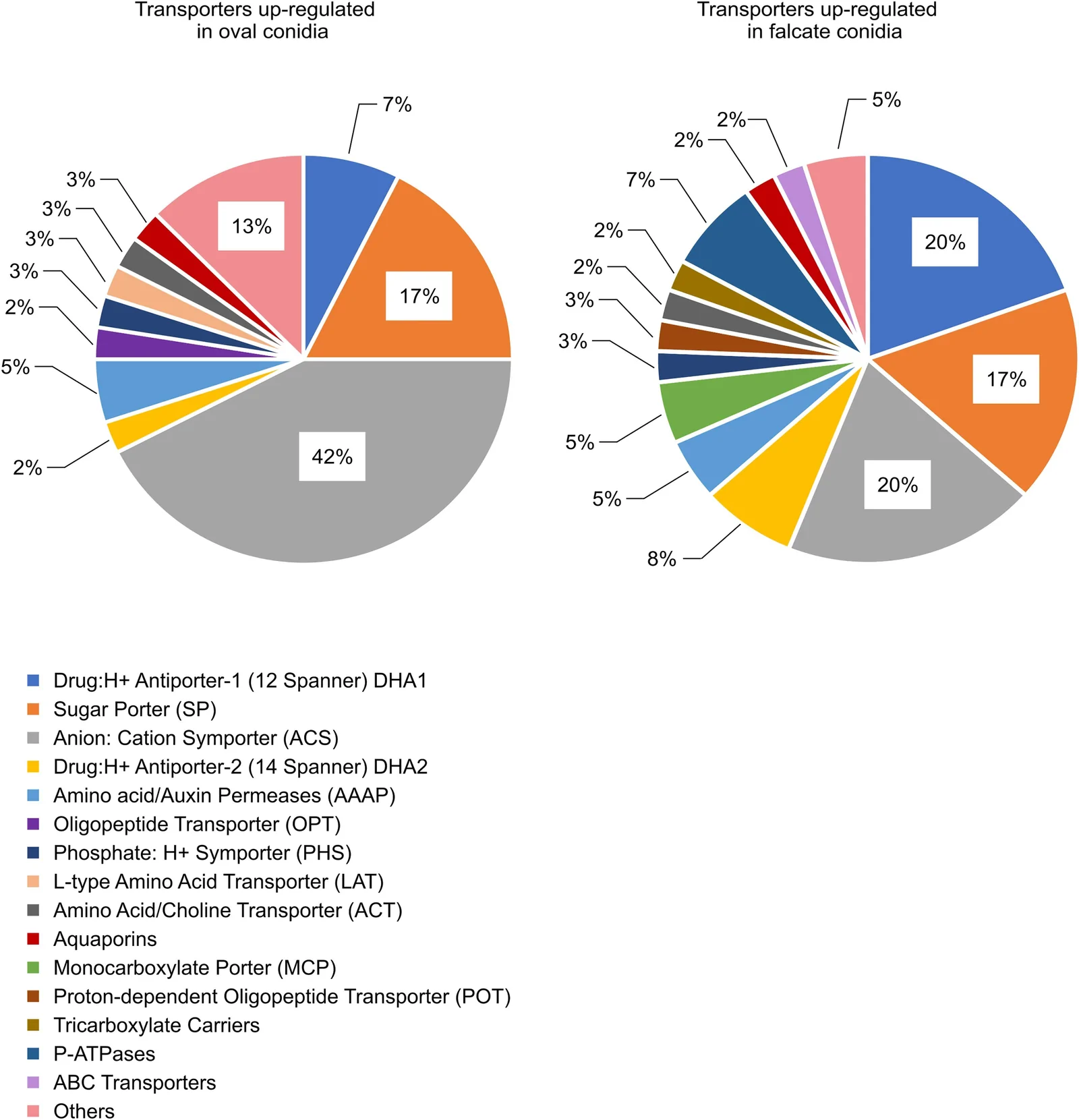
Differentially expressed transporter genes showing induced expression during early leaf infection. Genes were identified as differentially expressed if they had an adjusted P-value (P_adj_) of <0.05 and log2 values of fold expression changes were either ≥ 1 for up-regulated or ≤−1 for down-regulated genes. Transporter gene families with elevated expression in falcate conidia and oval conidia, identified among the top 40 expressed genes during early leaf infections.

#### Genes encoding CAZymes

Microorganisms produce a diverse repertoire of CAZymes involved in the synthesis, modification, and degradation of complex carbohydrates (Wardman and Withers 2024). These enzymes hydrolyze major plant cell wall polymers such as cellulose, hemicellulose, and pectin, enabling the utilization of plant biomass as a nutrient source. CAZymes are organized into distinct families based on protein sequence similarity and conserved three-dimensional structural folds (Barrett et al. 2020a).

In total, 663 CAZymes were annotated in the new genome version 5, out of which 558 are differentially expressed in at least one pairwise comparison (O_vs_M, F_vs_M, LO_vs_O, O5_vs_O, O16_vs_O, LF_vs_F, F5_vs_F, F16_vs_F, LF_vs_LO; Dataset S7). This is an increase from the previously annotated 467 CAZymes, highlighting the complexity and diversity of CAZyme regulation in this fungus (Baroncelli et al. 2016). To reveal CAZyme families showing a high level of regulation during development and pathogenicity, the Top 100 variable genes were analyzed in detail (Fig. 7, a and b; Supplementary Fig. 11). In general, similar patterns to transporter-encoding genes can be observed: besides CAZmyes regulated in an opposite way in oval and falcate conidia, there are several showing a general up-regulation during early leaf infection stage in both spore types. That group might stand for a general adaptation of the fungus from saprophytic to pathogenic growth, in which predominantly complex substrates from plants are used for nutrition. Among the families showing strict spore type specificity there were, for example, Carbohydrate Esterase family CE16 (acetylesterase) uniquely up-regulated in falcate conidia, whereas Auxiliary Activity famiy AA12 + AA8 (oxidoreductase) was specifically induced in oval conidia. The most striking distinction between spore types was observed in the Auxiliary Activities (AA) family (Fig. 7, Supplementary Fig. 12). In falcate conidia, members of the AA9 family (lytic polysaccharide monooxygenases with cellulase and xylanase activities) were strongly up-regulated, alongside higher expression of Glycoside Hydrolase family GH10 cellulase/xylanase genes (Supplementary Fig. 12). These results suggest that falcate spores may be primed for the rapid breakdown of cellulose- and hemicellulose-rich plant cell walls, facilitating host tissue penetration and enabling nutrient release during the early necrotrophic phase of infection. In contrast, oval conidia exhibited elevated expression of Polysaccharide Lyase family PL3 (pectate lyase) during early developmental stages (5 h and 16 h; Supplementary Fig. 13). The indicated specialization for the degradation of pectin, an abundant component of the middle lamella, could support more targeted and localized modification of host cell walls by oval conidia, potentially aiding in adhesion or subtle wall loosening.

**Fig. 7.**
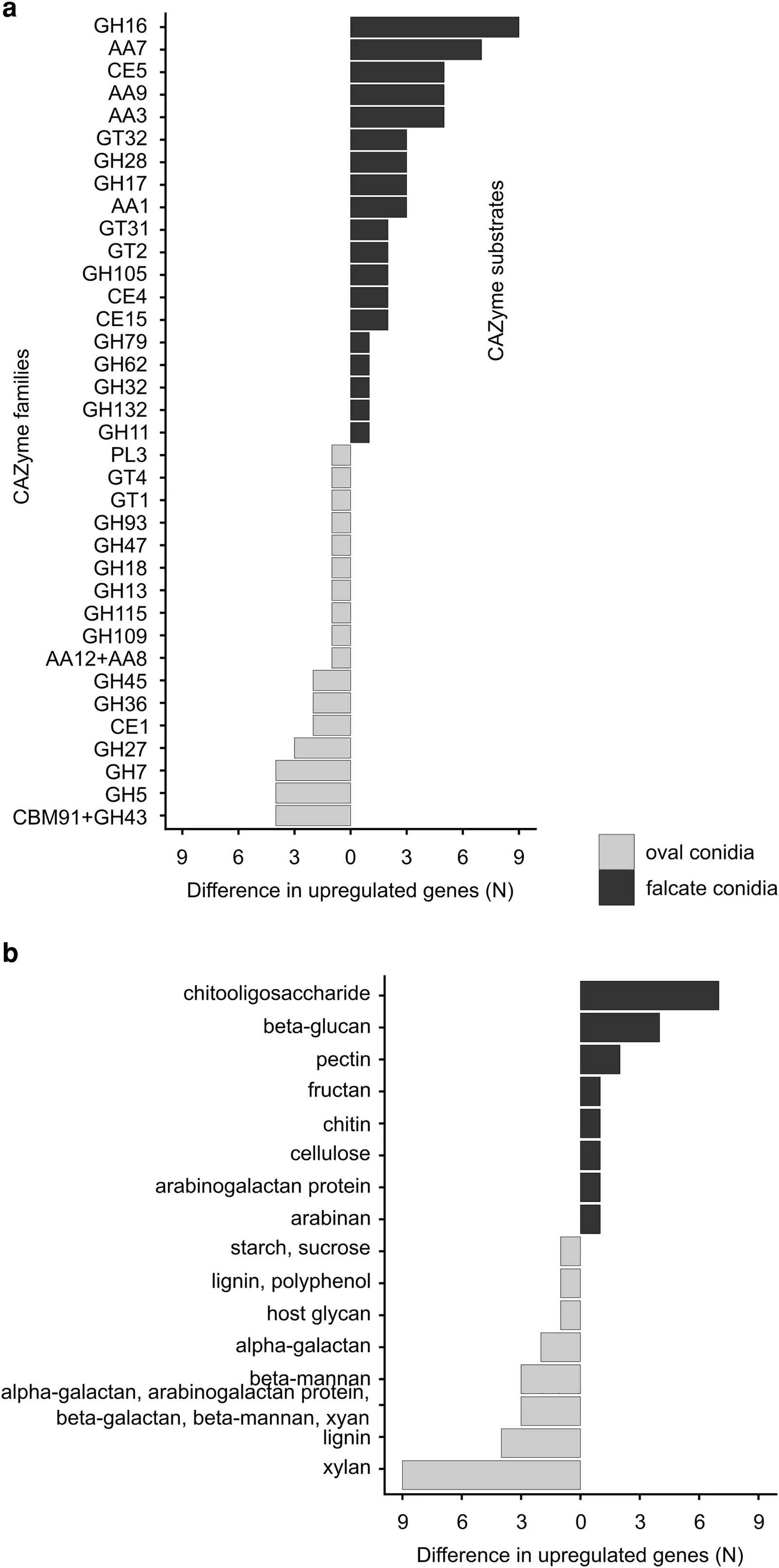
Most variable differentially expressed carbohydrate-active enzymes (CAZymes) in *C. graminicola* asexual spore types during leaf infection. As reference condition served freshly harvested conidia of the corresponding spore type. a) Distribution of CAZyme families and (b) corresponding predicted CAZyme substrate categories associated, up-regulated in oval and falcate conidia. Identification of the top 100 variable genes was performed using iDEP.96 (Ge et al. 2018) on the basis of differentially expressed genes identified (DEseq).

#### Genes encoding regulators of development

In fungi, the regulatory proteins coordinating crucial processes such as growth, spore formation (conidiogenesis), morphological differentiation, reproduction, and development of specialized structures like fruiting bodies and plant surface penetrating structures, are highly conserved (Nolting and Pöggeler 2006; Wong Sak Hoi and Dumas 2010; Lichius and Lord 2014; Turrà et al. 2014). For this study, we verified the presence of 122 conserved regulators of development using BLAST analysis based on *Sordaria macrospora and Aspergillus flavus* NRRL3357 protein sequences (Dataset S8; Teichert et al. 2020; Cho et al. 2022). Most developmental genes did not show a high level of regulation at the different timepoints investigated. Genes which showed a regulation pattern, however, exhibited a general down-regulation in freshly harvested spores and an induction of expression during the progression of vegetative development and pathogenicity.

Among the major regulated genes, the ones encoding enzymes in the melanin biosynthesis were up-regulated in both spore types during leaf infection (Supplementary Fig. 14). This aligns with previous findings that melanin functions are an important virulence factor in plant-pathogenic fungi, facilitating early host penetration, stress adaptation, and evasion of plant defenses (Howard and Valent 1996; Li et al. 2024). Similarly, we observed induction of Rho4-like GTPase, which plays a crucial role in various cellular processes, including β-1,3-glucan synthesis, cell wall integrity, growth of vegetative hyphae, conidiation, infection structure differentiation, and is also required for full virulence, in both spore types during early leaf infections (Oliveira-Garcia et al. 2022). For fungal NADPH oxidase complexes (NOX), we found very distinct expression patterns: whereas subunits required for the assembly of the NOX1 complex (catalytic subunit Nox1 (COGRA5_02814), NoxD regulator (COGRA5_05373)) showed a strong regulation depending on the developmental stage or spore type, the catalytic subunit of the NOX2 complex, Nox2 (COGRA5_09929), showed hardly any gene regulation. This is interesting, since both complexes are associated with very different developmental processes in fungi, albeit both are generating signaling competent reactive oxygen species (Siegmund et al. 2015; Li et al. 2022): NOX1 was associated in with successful germling fusion, vegetative growth and sexual development, whereas NOX2 is required for ascospore germination and chemotropic sensing of root derived class III peroxidases in several fungi. In interactions with the host, however, both NOX complexes serve the penetration of the plant surface (Cano-Domínguez et al. 2008; Kayano et al. 2013; Ryder et al. 2013; Dirschnabel et al. 2014; Marschall et al. 2016; Nordzieke et al. 2019a; Rudolph et al. 2024). Thus, one might expect differential patterns of *Cgnox1* and *Cgnox2* gene expressions in the different samples investigated. The absence of a clear transcript regulation for *Cgnox2* might thus indicate that the activity of the NOX2 complex is not regulated primarily by gene expression changes, but by the assembly of the complex components.

#### SM biosynthesis genes

SMs are low molecular weight organic compounds produced by fungi, plants and bacteria. In contrast to primary metabolites essential for the organisms’ survival, SMs are produced in specific environmental and developmental conditions to improve ecological fitness (Bills and Gloer 2016; Macheleidt et al. 2016). Primary metabolites originating from central metabolic pathways serve as essential building blocks together with Acyl-CoAs and amino acids (Macheleidt et al. 2016; Keller 2019). Fungal SMs are chemically diverse and fall into four major classes: polyketides, terpenoids, phenolics, and non-ribosomal peptides (Pusztahelyi et al. 2015). Genes responsible for SM biosynthesis are typically organized in contiguous order as biosynthetic gene clusters (BGC; Keller 2019). Regulation of BGCs is closely related to the ecological context and infection stage, underlining the adaptive significance of secondary metabolism during pathogenesis (Lysøe et al. 2011; Keller 2019).

Based on the *C. graminicola* genome sequence V5, gene candidates belonging to SM biosynthesis clusters were predicted using antiSMASH version fungiSMASH 7.0.0 (Blin et al. 2021). Among these, 466 genes are differentially regulated during various developmental stages and early stages of maize leaf infection 1 dpi (Dataset S9). Notably, 193 SM BGCs displayed a decreased expression in freshly harvested samples of both falcate and oval conidia (Supplementary Fig. 15). Conversely, 133 BGCs exhibited induction during leaf infection in both spore types. Notably, the fusaridione A cluster, which was originally identified in *Fusarium heterosporumin,* was induced upon host interaction, aligning with previous reports (Kakule et al. 2013). A similar pattern is displayed by the depudecin BGC. This cluster enables biosynthesis of the histone deacetylase inhibitor depudecin, which contributes to virulence in *Alternaria brassicicola* (Wight et al. 2009; Pedras and Park 2015). Additionally, the BGC of three antimitotic mycotoxins, phomopsins A, B, and E display increased expression during host interaction, similarly to observations from *Phomopsis leptostromiformis* (Hamel 1992; Grimley et al. 2007; Izuchi et al. 2011).

## Discussion

In this study, we performed a comprehensive RNA-seq analysis based on genome resequencing of our laboratory strain of the corn anthracnose fungus *C. graminicola* which is derived from the original isolate CgM2/M1.001. We focused on the elucidation of spore type-specific gene expression patterns of critical stages in development and early leaf infection of oval and falcate conidia and their derived hyphae. For data interpretation, we concentrated on six functional gene groups, comprising genes relevant as TFs, effectors, transporter proteins, CAZymes, developmental regulators, and as parts of secondary metabolite gene clusters. Overall, we found gene expression changes of both conidia types regarding germination and germling stages, along with a general increase in gene expression levels observed during early leaf infections. In comparison to mycelium samples, freshly harvested oval and falcate conidia showed clear differences in gene expression patterns, with overlaps between the two conidia types but also spore type-specific gene expression changes are evident (Fig. 2). Similarly, we found that during early maize leaf infection the top variable genes encoding effector proteins, transporters, and CAZymes show uniform expression patterns, reflecting the general adaptation to a host environment. At the same time, several genes are specifically regulated in oval and falcate conidia, some with even opposite expression patterns (up-regulation in oval conidia and down-regulation in falcate conidia and vice versa), pointing to probable spore type-specific processes during host interactions (Supplementary Figs. 8 and 10, and 11 File 1). Together, these findings indicate that oval and falcate-shaped spores express a core gene set responsible for their identity as conidia, clearly different from vegetative mycelium. On the other hand, both conidia types have an individual signature, which is maintained also during early maize leaf infection. These findings support earlier findings from phenotypic investigations, in which oval and falcate conidia were shown to undergo specific developmental processes and explore unique infection strategies meanwhile interacting with host leaves (Nordzieke et al. 2019b). Altogether, this study provides a comprehensive overview about gene expression changes in vegetative development and during early leaf interaction of oval and falcate conidia, forming the basis for spore type-specific development and virulence of *C. graminicola*.

In previous studies, genome sequencing in combination with RNA-seq was used to understand critical factors determining the symptoms developed by closely related plant pathogens. For example, in *Zymoseptoria*, a genus of plant-pathogenic fungi specialized in the infection of grasses, comparison of the genomes of four different species identified species-specific orphan genes and genes encoding small, secreted proteins with putative functions for virulence and host specificity (Grandaubert et al. 2015). Furthermore, RNA-seq analysis of eight *Fusarium fujikuroi* isolates revealed a correlation between the formation of SMs and two distinct pathotypes on rice. In this case, gibberellic acid (GA) biosynthesis is positively correlated with typical symptom development of *bakanae* disease like hyper-elongated seedlings, while fumosin-generating strains showed stunting and early withering of infected seedlings (Niehaus et al. 2017). Similarly, RNA-seq can give important hints to the regulation of plant defense responses during pathogenic interactions. For example, comparison of gene expression profiles of soybeans infected with non-pathogenic and pathogenic strains of *Fusarium oxysporum* revealed high numbers and magnitudes of differentially expressed genes (HDEGs) in the plant comparing both infection scenarios (Lanubile et al. 2015). In all these examples, different isolates of the same pathogenic species or closely related species were analyzed, having isolate-specific or species-specific genetic information encoded in their genomes, respectively. Whereas comparable studies are numerous, distinct transcriptomes of asexual spore types of a single species with the same genetic information are underexplored. In one case, the transcriptomes of freshly harvested microconidia and macroconidia of the rice blast fungus *Magnaporthe oryzae* were analyzed, providing evidence that only seven genes are specifically expressed in microconidia and eight additional genes show increased expression in macroconidia after harvest (Zhang et al. 2014). In our study, the numbers of differentially regulated genes identified in freshly harvested oval and falcate conidia of *C. graminicola* were much higher (Fig. 2): compared to mycelium samples, oval and falcate conidia showed increased expression of 1,483 genes in common and, additionally, similar numbers of up-regulated spore type-specific genes (oval conidia: 1,446, falcate conidia: 1,771). We observed a similar pattern when comparing down-regulated genes respective to mycelium samples: in both conidia types, 1,801 genes are down-regulated, but we observed >1,000 genes specifically for oval (1,168) and falcate conidia (1,408). Bearing in mind that the genome of *C. graminicola* encodes 15,481 genes, we observed roughly 30% of the genes up-regulated and 28% of genes down-regulated in freshly harvested oval and falcate conidia, underpinning the metabolic activity of both spore types. Monitoring later stages of development, we observed rapid transcriptional changes in both spore types after 5 h, although only oval conidia showed germination under the conditions monitored and falcate conidia remained dormant. These surprising observations, however, are in line with a previous report regarding transcriptional activity of dormant *Aspergillus* conidia, providing evidence that fungal conidia are able to react to environmental signals, shaping the transcripts and thus the later behavior after dormancy breaking (Wang et al. 2021).

Apart from general gene expression changes, we identified numerous genes with similar, specific or even opposite regulations in oval and falcate conidia, of which we further analyzed a subset using qRT-PCR (Fig. 3). For instance, COGRA5_11040, encoding an Aspzincin_M35 metallopeptidase, is highly induced in both oval and falcate conidia during early leaf infection (1 dpi). Since metalloproteinases degrade proteins in host tissues and increase thereby the hosts’ susceptibility to disease, this result might indicate that oval and falcate conidia use the same strategy for masking their presence (Huang et al. 2020; Wang et al. 2024). In contrast, Lysine-domain-containing proteins (LysMs) were mainly induced during leaf infection with falcate conidia, but to a lesser extent with oval conidia (Dataset S10). However, the LysM encoding gene COGRA5_04487 even shows an opposite regulation. Fungal LysM proteins are effectors and play crucial roles in regulating development, maintaining cell wall integrity by binding chitin, and contributing to pathogenicity by suppressing host defenses and facilitating host colonization (Muraosa et al. 2019; Dubey et al. 2020). In contrast to those findings, most of the *TauD* genes present in *C. graminicola* genome are highly expressed during oval conidia leaf infection, but not in falcate conidia (Fig. 3; Dataset S3). TauD proteins enable fungi to survive in nutrient-deprived conditions by utilizing taurine and xanthine as nutrient sources (Eichhorn et al. 1997; Montero-Morán et al. 2007). This is consistent with our previous results that oval conidia are able to survive and germinate in nutrient-deprived conditions, but falcate conidia are not (Nordzieke et al. 2019b; Rudolph et al. 2025b). Intriguingly, nutrient starvation induced by cultivation media or the inoculation on intact plant surfaces induce fusion of oval conidia-derived germlings, which give rise to hyphopodia formation for plant penetration (Nordzieke et al. 2019b). *TauD* genes therefore could contribute to successful germination and germling fusion during colony formation and the pathogenicity of *C. graminicola*.

Additionally, our RNA-seq analysis indicated that the two spore types of *C. graminicola* use distinct gene repertoires to adapt to the shift from saprotrophic growth to pathogenic interaction with the plant. For example, CAZymes and transporters, which are required for nutrient acquisition and uptake, comprise family members with a specific up-regulation during leaf infections in both spore types (Supplementary Figs. 10 and 11 File 1). At the same time, there are several CAZyme and transporter-encoding genes, which are oppositely regulated in oval and falcate conidia, meaning that gene up-regulation in oval conidia samples parallels down-regulation in falcate conidia. Such regulation indicates that these genes might be vital for nutrition of a distinct spore type. For example, the CAZyme families AA9 and GH10 are induced in falcate conidia during early leaf infections. Together, these enzymes enable rapid breakdown of cellulose- and hemicellulose-rich plant cell walls and facilitate tissue penetration and nutrient release in early infection (Bradley et al. 2022; Supplementary Fig. 10). At the same time, falcate conidia show an elevated number of DHA transporters among the top regulated genes during early leaf infections, indicating an increased capacity for the detoxification of host defense metabolites (Cavalheiro et al. 2018). In contrast, oval conidia express oligopeptide transporters (OPT) and L-type amino acid transporters (LAT), which are potentially involved in organic nitrogen uptake from the host (Reuß and Morschhäuser 2006; Kantipudi and Fotiadis 2021; Fig. 6). Additionally, elevated expression of PL3 (pectate lyase) during early vegetative development (5 h and 16 h) was observed, which degrades pectin in the middle lamella of plant cells, supporting adhesion, controlled wall loosening, and early biotrophic establishment without extensive damage (Fig. 7, Supplementary Fig. 12; Yang et al. 2018). Focusing on expressed effector genes, we observed the same general pattern: several effector encoding genes were induced upon the initiation of host leaf infections in oval and falcate conidia (Supplementary Figs. 8 and 9 Files 1), indicating a general role in the interaction with the host. In addition, there are some effector genes, which are differentially regulated when we compare gene expressions between early leaf infection of falcate and oval conidia (Fig. 5, Supplementary Table 4). One of these, COGRA5_10559, was previously characterized as necrotrophy-associated, switch-specific effector in *C. graminicola* falcate conidia (Kleemann et al. 2012; Oome and Van den Ackerveken 2014; Torres et al. 2016; Seidl and Van den Ackerveken 2019). Interpreting these data, it is however important to keep in mind the different strategies for leaf infection explored by both conidia types: oval conidia form germling networks on leaves, whereas falcate conidia form appressoria from short germ tubes. Independent of the infection process, both spore types are therefore in different physiological stages, which might explain some of the transcriptomal changes seen during early leaf infections. Future studies will have to reveal whether the conidia-specific gene expression patterns seen have consequences for the maize infection processes induced by *C. graminicola* oval and falcate conidia.

## Supplementary Material

jkag254_Supplementary_Data

## Data Availability

The data underlying this article are available in the European Nucleotide Archive (ENA; https://www.ebi.ac.uk/ena/browser/home) and can be accessed under the project numbers PRJEB101887 (genome resequencing and assembly), PRJEB104144 (RNA-seq). The annotated genome files including genome assembly, annotation, and sequences for each contig have been added as supplementary information (Supplementary file 3-17). For submission of supplementary datasets, Supplementary File 2-17, GSA FigShare portal was used (https://doi.org/10.25387/g3.33365940). That submission contains the following datasets: Dataset S1: Annotation of protein coding genes of *Colletotrichum graminicola* M1.001 (CgM2 wildtype strain) with new gene IDs (COGRA5); Dataset S2: RNA-seq data of all the *Colletotrichum graminicola* M1.001 (CgM2 wildtype strain) genes labeled with different locus tags; Dataset S3: Expression of Taurine catabolism dioxygenase (TauD) genes in *Colletotrichum graminicola* M1.001 (CgM2 wildtype strain); Dataset S4: RNA-seq data of predicted TFs in *Colletotrichum graminicola* M1.001 (CgM2 wildtype strain), identified using data from 312 predicted TF genes in *Neurospora crassa* OR74A (Carrillo et al. 2017); Dataset S5: RNA-seq data of predicted effector genes in *Colletotrichum graminicola* M1.001 (CgM2 wildtype strain); Dataset S6: RNA-seq data of predicted membrane transport proteins in *Colletotrichum graminicola* M1.001 (CgM2 wildtype strain); Dataset S7: RNA-seq data of predicted CAZymes in *Colletotrichum graminicola* M1.001 (CgM2 wildtype strain); Dataset S8: RNA-seq data of predicted developmental genes in *Colletotrichum graminicola* M1.001 (CgM2 wildtype strain), identified using data based on *Sordaria macrospora* and *Aspergillus flavus* NRRL3357 protein sequences (Teichert et al. 2020; Cho et al. 2022); Dataset S9: RNA-seq data of predicted SM biosynthesis clusters in *Colletotrichum graminicola* M1.001 (CgM2 wildtype strain), which was predicted using antiSMASH version fungiSMASH 7.0.0 (Blin et al. 2021). Dataset S10: Expression of LysM candidates (CBM 50) in *Colletotrichum graminicola* M1.001 (CgM2 wildtype strain); File S2: source code for perl scripts; File S3: sequence of COGRA5_contig_1; File S4: sequence of COGRA5_contig_2; File S5: sequence of COGRA5_contig_3; File S6: sequence of COGRA5_contig_4; File S7: sequence of COGRA5_contig_5; File S8: sequence of COGRA5_contig_6; File S9: sequence of COGRA5_contig_7; File S10: sequence of COGRA5_contig_8; File S11: sequence of COGRA5_contig_9; File S12: sequence of COGRA5_contig_10; File S13: sequence of COGRA5_contig_11; File S14: sequence of COGRA5_contig_12; File S15: sequence of COGRA5_contig_13; File S16: genome annotation; File S17: genome sequence; graphical abstract: graphical abstract. Supplemental material available at G3 online.

## References

[jkag254-B1] Aliyeva-Schnorr L, Heise NV, Csuk R, Deising HB. 2025. Redox status-selective imaging of iron in vegetative and pathogenic fungal cells using fluorescent dyes synthesized via simple chemical reactions. Mol Plant Microbe Interact. 38:50–55. 10.1094/MPMI-09-24-0111-SC. 39869149

[jkag254-B2] Assaf JC et al 2024. Comparative review on the production and purification of bioethanol from biomass: a focus on corn. Processes. 12:1001. 10.3390/pr12051001.

[jkag254-B3] Baroncelli R et al 2016. Gene family expansions and contractions are associated with host range in plant pathogens of the genus *Colletotrichum*. BMC Genomics. 17:555. 10.1186/s12864-016-2917-6. PMC4974774 27496087

[jkag254-B4] Baroncelli R et al 2017. The *Colletotrichum acutatum* species complex as a model system to study evolution and host specialization in plant pathogens. Front Microbiol. 8:2001. 10.3389/fmicb.2017.02001. PMC5641571 29075253

[jkag254-B5] Barrett K et al 2020a. Fungal secretome profile categorization of CAZymes by function and family corresponds to fungal phylogeny and taxonomy: example *Aspergillus* and *Penicillium*. Sci Rep. 10:5158. 10.1038/s41598-020-61907-1. PMC7083838 32198418

[jkag254-B6] Barrett K, Hunt CJ, Lange L, Meyer AS. 2020b. Conserved unique peptide patterns (CUPP) online platform: peptide-based functional annotation of carbohydrate active enzymes. Nucleic Acids Res. 48:W110–W115. 10.1093/nar/gkaa375. PMC7319580 32406917

[jkag254-B7] Becerra S et al 2023. Chromosome-level analysis of the *Colletotrichum graminicola* genome reveals the unique characteristics of core and minichromosomes. Front Microbiol. 14:. 10.3389/fmicb.2023.1129319. PMC10076810 37032845

[jkag254-B8] Belisário R, Robertson AE, Vaillancourt L. 2022. Maize anthracnose stalk rot in the genomic era. Plant Dis. 106:2281–2298. 10.1094/PDIS-10-21-2147-FE. 35291814

[jkag254-B9] Bergstrom GC, Nicholson RL. 1999. The biology of corn anthracnose: knowledge to exploit for improved management. Plant Dis. 83:596–608. 10.1094/PDIS.1999.83.7.596. 30845609

[jkag254-B10] Bettini PP et al 2012. Identification and characterization of GEO1, a new class II hydrophobin from *Geosmithia* spp. Can J Microbiol. 58:965–972. 10.1139/w2012-069. 22803587

[jkag254-B11] Bills GF, Gloer JB. 2016. Biologically active secondary metabolites from the fungi. Microbiol Spectrum. 4:FUNK-0009-2016. 10.1128/microbiolspec.funk-0009-2016. 27809954

[jkag254-B12] Blin K et al antiSMASH 6.0: improving cluster detection and comparison capabilities. 2021. Nucleic Acids Res. 49:W29–W35. 10.1093/nar/gkab335. PMC8262755 33978755

[jkag254-B13] Bork P . 1993. Hundreds of ankyrin-like repeats in functionally diverse proteins: mobile modules that cross phyla horizontally? Proteins. 17:363–374. 10.1002/prot.340170405. 8108379

[jkag254-B14] Boufleur TR et al 2022. Comparative transcriptomic provides novel insights into the soybean response to *Colletotrichum truncatum* infection. Front Plant Sci. 13:1046418. 10.3389/fpls.2022.1046418. PMC9732023 36507428

[jkag254-B15] Bradley EL et al 2022. Secreted glycoside hydrolase proteins as effectors and invasion patterns of plant-associated fungi and oomycetes. Front Plant Sci. 13:853106. 10.3389/fpls.2022.853106. PMC8960721 35360318

[jkag254-B16] Brůna T et al 2021. BRAKER2: automatic eukaryotic genome annotation with GeneMark-EP+ and AUGUSTUS supported by a protein database. NAR Genom Bioinform. 3:lqaa108. 10.1093/nargab/lqaa108. PMC7787252 33575650

[jkag254-B17] Cannon PF, Damm U, Johnston PR, Weir BS. 2012. *Colletotrichum*: current status and future directions. Stud Mycol. 73:181–213. 10.3114/sim0014. PMC3458418 23136460

[jkag254-B18] Cano-Domínguez N, Álvarez-Delfín K, Hansberg W, Aguirre J. 2008. NADPH oxidases NOX-1 and NOX-2 require the regulatory subunit NOR-1 to control cell differentiation and growth in *Neurospora crassa*. Eukaryot Cell. 7:1352–1361. 10.1128/ec.00137-08. PMC2519770 18567788

[jkag254-B19] Cantarel BL et al 2008. MAKER: an easy-to-use annotation pipeline designed for emerging model organism genomes. Genome Res. 18:188–196. 10.1101/gr.6743907. PMC2134774 18025269

[jkag254-B20] Carrillo AJ et al 2017. Functional profiling of transcription factor genes in *Neurospora crassa*. G3 (Bethesda). 7:2945–2956. 10.1534/g3.117.043331. PMC5592922 28696922

[jkag254-B21] Cavalheiro M, Pais P, Galocha M, Teixeira MC. 2018. Host-pathogen interactions mediated by MDR transporters in fungi: as pleiotropic as it gets!. Genes (Basel). 9:332. 10.3390/genes9070332. PMC6071111 30004464

[jkag254-B22] Chaky J, Anderson K, Moss M, Vaillancourt L. 2001. Surface hydrophobicity and surface rigidity induce spore germination in *Colletotrichum graminicola*. Phytopathology. 91:558–564. 10.1094/PHYTO.2001.91.6.558. 18943944

[jkag254-B23] Cho HJ et al 2022. Regulation of conidiogenesis in *Aspergillus flavus*. Cells. 11:2796. 10.3390/cells11182796. PMC9497164 36139369

[jkag254-B24] Dahl SG, Sylte I, Ravna AW. 2004. Structures and models of transporter proteins. J Pharmacol Exp Ther. 309:853–860. 10.1124/jpet.103.059972. 14988415

[jkag254-B25] Damm U et al The *Colletotrichum dracaenophilum*, *C. magnum* and *C. orchidearum* species complexes. Stud Mycol. 2019; 92: 1–46. 10.1016/j.simyco.2018.04.001. PMC6030544 29997400

[jkag254-B26] Dean R et al 2012. The top 10 fungal pathogens in molecular plant pathology. Mol Plant Pathol. 13:414–430. 10.1111/j.1364-3703.2011.00783.x. PMC6638784 22471698

[jkag254-B27] De Silva D et al 2017. Life styles of *Colletotrichum* species and implications for plant biosecurity. Fungal Biol Rev. 31:155–168. 10.1016/j.fbr.2017.05.001.

[jkag254-B28] Dirschnabel DE et al 2014. New insights into the roles of NADPH oxidases in sexual development and ascospore germination in *Sordaria macrospora*. Genetics. 196:729–744. 10.1534/genetics.113.159368. PMC3948803 24407906

[jkag254-B29] Dubey M et al 2020. Lysm proteins regulate fungal development and contribute to hyphal protection and biocontrol traits in *Clonostachys rosea*. Front Microbiol. 11:679. 10.3389/fmicb.2020.00679. PMC7176902 32373095

[jkag254-B30] Eichhorn E, van der Ploeg JR, Kertesz MA, Leisinger T. 1997. Characterization of α-ketoglutarate-dependent taurine dioxygenase from *Escherichia coli*. J Biol Chem. 272:23031–23036. 10.1074/jbc.272.37.23031. 9287300

[jkag254-B31] Eisermann I et al 2019. Two genes in a pathogenicity gene cluster encoding secreted proteins are required for appressorial penetration and infection of the maize anthracnose fungus *Colletotrichum graminicola*. Environ Microbiol. 21:4773–4791. 10.1111/1462-2920.14819. 31599055

[jkag254-B32] Forgey WM, Blanco MH, Loegering WQ. 1978. Differences in pathological capabilities and host specificity of *Colletotrichum graminicola* on *Zea mays*. Plant Dis Rep. 62:573–576.

[jkag254-B33] Frey TJ et al 2011. Fitness evaluation of Rcg1, a locus that confers resistance to *Colletotrichum graminicola* (Ces.) GW Wils. Using near-isogenic maize hybrids. Crop Sci. 51:1551–1563. 10.2135/cropsci2010.10.0613.

[jkag254-B34] Ge SX, Son EW, Yao R. 2018. iDEP: an integrated web application for differential expression and pathway analysis of RNA-Seq data. BMC Bioinformatics. 19:534. 10.1186/s12859-018-2486-6. PMC6299935 30567491

[jkag254-B35] Goedhart J, Luijsterburg MS. 2020. VolcaNoseR is a web app for creating, exploring, labeling and sharing volcano plots. Sci Rep. 10:20560. 10.1038/s41598-020-76603-3. PMC7689420 33239692

[jkag254-B36] Gomes S, Azevedo-Nogueira F, Martins-Lopes P. 2021. Editorial comments to the special issue:“*Colletotrichum* spp. on Fruit Crops—State of the art, perspectives and drawbacks”. Pathogens. 10:478. 10.3390/pathogens10040478. PMC8071184 33920756

[jkag254-B37] Grabherr MG et al 2011. Full-length transcriptome assembly from RNA-Seq data without a reference genome. Nat Biotechnol. 29:644–652. 10.1038/nbt.1883. PMC3571712 21572440

[jkag254-B38] Grandaubert J, Bhattacharyya A, Stukenbrock EH. 2015. RNA-seq-based gene annotation and comparative genomics of four fungal grass pathogens in the genus *Zymoseptoria* identify novel orphan genes and species-specific invasions of transposable elements. G3 (Bethesda). 5:1323–1333. 10.1534/g3.115.017731. PMC4502367 25917918

[jkag254-B39] Grimley JS et al 2007. The enantioselective synthesis of phomopsin B. Angew Chem Int Ed Engl. 46:8157–8159. 10.1002/anie.200702537. PMC3290521 17886312

[jkag254-B40] Gu X et al 2024. Plant immunity suppression by an β-1, 3-glucanase of the maize anthracnose pathogen *Colletotrichum graminicola*. BMC Plant Biol. 24:339. 10.1186/s12870-024-05053-0. PMC11046878 38671375

[jkag254-B41] Gupta C, Salgotra RK, Damm U, Rajeshkumar KC. 2022. Phylogeny and pathogenicity of *Colletotrichum lindemuthianum* causing anthracnose of *Phaseolus vulgaris* cv. Bhaderwah-Rajmash from northern Himalayas, India. 3 Biotech. 12:169. 10.1007/s13205-022-03216-0. PMC9279525 35845112

[jkag254-B42] Hamel E . 1992. Natural products which interact with tubulin in the vinca domain: maytansine, rhizoxin, phomopsin A, dolastatins 10 and 15 and halichondrin B. Pharmacol Ther. 55:31–51. 10.1016/0163-7258(92)90028-X. 1287674

[jkag254-B43] Hoff KJ et al 2016. BRAKER1: unsupervised RNA-Seq-based genome annotation with GeneMark-ET and AUGUSTUS. Bioinformatics. 32:767–769. 10.1093/bioinformatics/btv661. PMC6078167 26559507

[jkag254-B44] Hoff KJ, Lomsadze A, Borodovsky M, Stanke M. 2019. Whole-genome annotation with BRAKER. In: Gene prediction: methods and protocols, Springer. p. 65–95. 10.1007/978-1-4939-9173-0_5 PMC6635606 31020555

[jkag254-B45] Howard RJ, Valent B. 1996. Breaking and entering: host penetration by the fungal rice blast pathogen *Magnaporthe grisea*. Annu Rev Microbiol. 50:491–512. 10.1146/annurev.micro.50.1.491. 8905089

[jkag254-B46] Huang A et al 2020. A M35 family metalloprotease is required for fungal virulence against insects by inactivating host prophenoloxidases and beyond. Virulence. 11:222–237. 10.1080/21505594.2020.1731126. PMC7051145 32079481

[jkag254-B47] Izuchi Y et al 2011. Synthesis and structural revision of phomopsin B, a novel polyketide carrying a 10-membered cyclic-ether ring. Org Lett. 13:3360–3363. 10.1021/ol2011117. 21615128

[jkag254-B48] Jaramillo VDA, Sukno SA, Thon MR. 2015. Identification of horizontally transferred genes in the genus *Colletotrichum* reveals a steady tempo of bacterial to fungal gene transfer. BMC Genomics. 16:2. 10.1186/1471-2164-16-2. PMC4320630 25555398

[jkag254-B49] John E, Singh KB, Oliver RP, Tan KC. 2021. Transcription factor control of virulence in phytopathogenic fungi. Mol Plant Pathol. 22:858–881. 10.1111/mpp.13056. PMC8232033 33973705

[jkag254-B50] Joshi R . 2018. A review on *Colletotrichum* spp. Virulence mechanism against host plant defensive factors. Med Plants Stud. 6:64–67. 10.22271/plants.2018.v6.i6b.02.

[jkag254-B51] Kakule TB, Sardar D, Lin Z, Schmidt EW. 2013. Two related pyrrolidinedione synthetase loci in *Fusarium heterosporum* ATCC 74349 produce divergent metabolites. ACS Chem Biol. 8:1549–1557. 10.1021/cb400159f. 23614392

[jkag254-B52] Kandil EE et al 2020. Potentials of organic manure and potassium forms on maize (*Zea mays* L.) growth and production. Sci Rep. 10:8752. 10.1038/s41598-020-65749-9. PMC7260245 32472061

[jkag254-B53] Kantipudi S, Fotiadis D. 2021. Yeast cell-based transport assay for the functional characterization of human 4F2hc-LAT1 and-LAT2, and LAT1 and LAT2 substrates and inhibitors. Front Mol Biosci. 8:676854. 10.3389/fmolb.2021.676854. PMC8193492 34124158

[jkag254-B54] Kayano Y et al 2013. Differential roles of NADPH oxidases and associated regulators in polarized growth, conidiation and hyphal fusion in the symbiotic fungus *Epichloë festucae*. Fungal Genet Biol. 56:87–97. 10.1016/j.fgb.2013.05.001. 23684536

[jkag254-B55] Keller NP . 2019. Fungal secondary metabolism: regulation, function and drug discovery. Nat Rev Microbiol. 17:167–180. 10.1038/s41579-018-0121-1. PMC6381595 30531948

[jkag254-B56] Kim D et al 2019. Graph-based genome alignment and genotyping with HISAT2 and HISAT-genotype. Nat Biotechnol. 37:907–915. 10.1038/s41587-019-0201-4. PMC7605509 31375807

[jkag254-B57] Kleemann J et al 2012. Sequential delivery of host-induced virulence effectors by appressoria and intracellular hyphae of the phytopathogen *Colletotrichum higginsianum*. PLoS Pathog. 8:e1002643. 10.1371/journal.ppat.1002643. PMC3320591 22496661

[jkag254-B58] Koren S et al 2017. Canu: scalable and accurate long-read assembly via adaptive *k*-mer weighting and repeat separation. Genome Res. 27:722–736. 10.1101/gr.215087.116. PMC5411767 28298431

[jkag254-B59] Lange M, Peiter E. 2020. Calcium transport proteins in fungi: the phylogenetic diversity of their relevance for growth, virulence, and stress resistance. Front Microbiol. 10:3100. 10.3389/fmicb.2019.03100. PMC6997533 32047484

[jkag254-B60] Lanubile A et al 2015. Transcriptome profiling of soybean (*Glycine max*) roots challenged with pathogenic and non-pathogenic isolates of *Fusarium oxysporum*. BMC Genomics. 16:1089. 10.1186/s12864-015-2318-2. PMC4687377 26689712

[jkag254-B61] Leite B . 1992. Production of mycosporine-alanine, acetic acid and uridine by cultures of Colletotrichum graminicola: their role in conidial maturation and development. Purdue University.

[jkag254-B62] Leite B, Nicholson RL. 1992. Mycosporine-alanine: a self-inhibitor of germination from the conidial mucilage of *Colletotrichum graminicola*. Exp Mycol. 16:76–86. 10.1016/0147-5975(92)90043-Q.

[jkag254-B63] Li H et al 2009. The sequence alignment/map format and SAMtools. Bioinformatics. 25:2078–2079. 10.1093/bioinformatics/btp352. PMC2723002 19505943

[jkag254-B64] Li R et al 2024. *Aabrm1*-mediated melanin synthesis is essential to growth and development, stress adaption, and pathogenicity in *Alternaria alternata*. Front Microbiol. 14:1327765. 10.3389/fmicb.2023.1327765. PMC10808324 38274752

[jkag254-B65] Li T, Kim D, Lee J. 2022. NADPH oxidase gene, *FgNoxD*, plays a critical role in development and virulence in *Fusarium graminearum*. Front Microbiol. 13:822682. 10.3389/fmicb.2022.822682. PMC8928025 35308369

[jkag254-B66] Lichius A, Lord KM. 2014. Chemoattractive mechanisms in filamentous fungi. Open Mycol J. 8:28–57. 10.2174/1874437001408010028.

[jkag254-B67] Liu F et al 2022. Updating species diversity of *Colletotrichum*, with a phylogenomic overview. Stud Mycol. 101:1–56. 10.3114/sim.2022.101.01. PMC9365046 36059896

[jkag254-B68] Livak KJ, Schmittgen TD. 2001. Analysis of relative gene expression data using real-time quantitative PCR and the 2^− ΔΔCT^ method. Methods. 25:402–408. 10.1006/meth.2001.1262. 11846609

[jkag254-B69] Love MI, Huber W, Anders S. 2014. Moderated estimation of fold change and dispersion for RNA-Seq data with DESeq2. Genome Biol. 15:550. 10.1186/s13059-014-0550-8. PMC4302049 25516281

[jkag254-B70] Lysøe E, Seong KY, Kistler HC. 2011. The transcriptome of *Fusarium graminearum* during the infection of wheat. Mol Plant Microbe Interact. 24:995–1000. 10.1094/MPMI-02-11-0038. 21585270

[jkag254-B71] Macheleidt J et al 2016. Regulation and role of fungal secondary metabolites. Annu Rev Genet. 50:371–392. 10.1146/annurev-genet-120215-035203. 27732794

[jkag254-B72] Manni M et al 2021. BUSCO update: novel and streamlined workflows along with broader and deeper phylogenetic coverage for scoring of eukaryotic, prokaryotic, and viral genomes. Mol Biol. 38:4647–4654. 10.1093/molbev/msab199. PMC8476166 34320186

[jkag254-B73] Marschall R, Siegmund U, Burbank J, Tudzynski P. 2016. Update on Nox function, site of action and regulation in *Botrytis cinerea*. Fungal Biol Biotechnol. 3:8. 10.1186/s40694-016-0026-6. PMC5611593 28955467

[jkag254-B74] Miranda VJ et al 2017. Comparative transcriptomic analysis indicates genes associated with local and systemic resistance to *Colletotrichum graminicola* in maize. Sci Rep. 7:2483. 10.1038/s41598-017-02298-8. PMC5449407 28559543

[jkag254-B75] Montero-Morán GM et al 2007. Purification and characterization of the Fe^II^-and α-ketoglutarate-dependent xanthine hydroxylase from *Aspergillus nidulans*. Biochemistry. 46:5293–5304. 10.1021/bi700065h. PMC2525507 17429948

[jkag254-B76] Mosavi LK, Minor DL Jr, Peng Z. 2002. Consensus-derived structural determinants of the ankyrin repeat motif. Proc Natl Acad Sci U S A. 99:16029–16034. 10.1073/pnas.252537899. PMC138559 12461176

[jkag254-B77] Muraosa Y, Toyotome T, Yahiro M, Kamei K. 2019. Characterisation of novel-cell-wall LysM-domain proteins LdpA and LdpB from the human pathogenic fungus *Aspergillus fumigatus*. Sci Rep. 9:3345. 10.1038/s41598-019-40039-1. PMC6399445 30833675

[jkag254-B78] Nie R et al 2017. Structure and function of the divalent anion/na^+^ symporter from *Vibrio cholerae* and a humanized variant. Nat Commun. 8:15009. 10.1038/ncomms15009. PMC5413979 28436435

[jkag254-B79] Niehaus EM et al 2017. Comparative genomics of geographically distant *Fusarium fujikuroi* isolates revealed two distinct pathotypes correlating with secondary metabolite profiles. PLoS Pathog. 13:e1006670. 10.1371/journal.ppat.1006670. PMC5675463 29073267

[jkag254-B80] Nishihara N . 1975. Two types of conidia of *Colletotrichum graminicol*a (Ces.) GW Wils. Formed on artificial media, and their pathogenicity. Jpn J Phytopathol. 41:171–175. 10.3186/jjphytopath.41.171.

[jkag254-B81] Nolting N, Pöggeler S. 2006. A STE12 homologue of the homothallic ascomycete *Sordaria macrospora* interacts with the MADS box protein MCM1 and is required for ascosporogenesis. Mol Microbiol. 62:853–868. 10.1111/j.1365-2958.2006.05415.x. 16999832

[jkag254-B82] Nordzieke DE et al 2019a. NADPH oxidase regulates chemotropic growth of the fungal pathogen *Fusarium oxysporum* towards the host plant. New Phytol. 224:1600–1612. 10.1111/nph.16085. 31364172

[jkag254-B83] Nordzieke DE et al 2019b. Specialized infection strategies of falcate and oval conidia of *Colletotrichum graminicola*. Fungal Genet Biol. 133:103276. 10.1016/j.fgb.2019.103276. 31550526

[jkag254-B84] Nordzieke DE . 2022. Hyphal fusions enable efficient nutrient distribution in *Colletotrichum graminicola* conidiation and symptom development on maize. Microorganisms. 10:1146. 10.3390/microorganisms10061146. PMC9231406 35744664

[jkag254-B85] O'Connell RJ et al 2012. Lifestyle transitions in plant pathogenic *Colletotrichum* fungi deciphered by genome and transcriptome analyses. Nat Genet. 44:1060–1065. 10.1038/ng.2372. PMC9754331 22885923

[jkag254-B86] Oliveira-Garcia E et al 2022. The small ras superfamily GTPase Rho4 of the maize anthracnose fungus *Colletotrichum graminicola* is required for β-1, 3-glucan synthesis, cell wall integrity, and full virulence. J Fungi (Basel). 8:997. 10.3390/jof8100997. PMC9604917 36294561

[jkag254-B87] Oome S, Van den Ackerveken G. 2014. Comparative and functional analysis of the widely occurring family of Nep1-like proteins. Mol Plant Microbe Interact. 27:1081–1094. 10.1094/MPMI-04-14-0118-R. 25025781

[jkag254-B88] Panaccione DG, Vaillancourt LJ, Hanau RM. 1989. Conidial dimorphism in *Colletotrichum graminicola*. Mycologia. 81:876–883. 10.1080/00275514.1989.12025677.

[jkag254-B89] Pedley KF, Walton JD. 2001. Regulation of cyclic peptide biosynthesis in a plant pathogenic fungus by a novel transcription factor. Proc Natl Acad Sci U S A. 98:14174–14179. 10.1073/pnas.231491298. PMC61187 11698648

[jkag254-B90] Pedras MSC, Park MR. 2015. Metabolite diversity in the plant pathogen *Alternaria brassicicola*: factors affecting production of brassicicolin A, depudecin, phomapyrone A and other metabolites. Mycologia. 107:1138–1150. 10.3852/15-054. 26297782

[jkag254-B91] Pusztahelyi T, Holb IJ, Pócsi I. 2015. Secondary metabolites in fungus-plant interactions. Front Plant Sci. 6:573. 10.3389/fpls.2015.00573. PMC4527079 26300892

[jkag254-B92] Reuß O, Morschhäuser J. 2006. A family of oligopeptide transporters is required for growth of *Candida albicans* on proteins. Mol Microbiol. 60:795–812. 10.1111/j.1365-2958.2006.05136.x. 16629678

[jkag254-B93] Revilla P et al 2022. Traditional foods from maize (*Zea mays* L.) in Europe. Front Nutr. 8:683399. 10.3389/fnut.2021.683399. PMC8780548 35071287

[jkag254-B94] Rudolph AY et al 2025a. PmaI-dependent pH gradients initiate inter-and intraspecies germling fusion in filamentous fungi. bioRxiv 2025.2006. 2024.661326. 10.1101/2025.06.24.661326

[jkag254-B95] Rudolph AY et al 2025b. Maize diterpenoid sensing via the Ste3 A-pheromone receptor guide oval conidia of *Colletotrichum graminicola* to host roots. Mol Plant Pathol. 26:e70155. 10.1111/mpp.70155. PMC12445352 40966175

[jkag254-B96] Rudolph AY, Schunke C, Nordzieke DE. 2024. Conserved perception of host and non-host signals via the a-pheromone receptor Ste3 in *Colletotrichum graminicola*. Front Fungal Biol. 5:1454633. 10.3389/ffunb.2024.1454633. PMC11491335 39435183

[jkag254-B97] Ryder LS et al 2013. NADPH oxidases regulate septin-mediated cytoskeletal remodeling during plant infection by the rice blast fungus. Proc Natl Acad Sci U S A. 110:3179–3184. 10.1073/pnas.1217470110. PMC3581893 23382235

[jkag254-B98] Samarakoon MC et al 2018. *Colletotrichum acidae* sp. nov. from northern Thailand and a new record of *C. dematium* on Iris sp. Mycosphere. 9:583–597. 10.5943/mycosphere/9/3/9.

[jkag254-B99] Schliebner I et al 2014. New gene models and alternative splicing in the maize pathogen *Colletotrichum graminicola* revealed by RNA-Seq analysis. BMC Genomics. 15:842. 10.1186/1471-2164-15-842. PMC4194422 25281481

[jkag254-B100] Seidl MF, Van den Ackerveken G. 2019. Activity and phylogenetics of the broadly occurring family of microbial Nep1-like proteins. Annu Rev Phytopathol. 57:367–386. 10.1146/annurev-phyto-082718-100054. 31283435

[jkag254-B101] Siegmund U, Marschall R, Tudzynski P. 2015. Bcnoxd, a putative ER protein, is a new component of the NADPH oxidase complex in *Botrytis cinerea*. Mol Microbiol. 95:988–1005. 10.1111/mmi.12869. 25402961

[jkag254-B102] Sonah H, Deshmukh RK, Bélanger RR. 2016. Computational prediction of effector proteins in fungi: opportunities and challenges. Front Plant Sci. 7:126. 10.3389/fpls.2016.00126. PMC4751359 26904083

[jkag254-B103] Stanke M, Diekhans M, Baertsch R, Haussler D. 2008. Using native and syntenically mapped cDNA alignments to improve *de novo* gene finding. Bioinformatics. 24:637–644. 10.1093/bioinformatics/btn013. 18218656

[jkag254-B104] Stanke M, Schöffmann O, Morgenstern B, Waack S. 2006. Gene prediction in eukaryotes with a generalized hidden Markov model that uses hints from external sources. BMC Bioinformatics. 7:62. 10.1186/1471-2105-7-62. PMC1409804 16469098

[jkag254-B105] Sukno SA, García VM, Shaw BD, Thon MR. 2008. Root infection and systemic colonization of maize by *Colletotrichum graminicola*. Appl Environ Microbiol. 74:823–832. 10.1128/AEM.01165-07. PMC2227703 18065625

[jkag254-B106] Talhinhas P, Baroncelli R. 2021. *Colletotrichum* species and complexes: geographic distribution, host range and conservation status. Fungal Divers. 110:109–198. 10.1007/s13225-021-00491-9.

[jkag254-B107] Team RC . 2024. R: a language and environment for statistical computing. R Foundation for Statistical Computing.

[jkag254-B108] Teichert I, Pöggeler S, Nowrousian M. 2020. *Sordaria macrospora*: 25 years as a model organism for studying the molecular mechanisms of fruiting body development. Appl Microbiol Biotechnol. 104:3691–3704. 10.1007/s00253-020-10504-3. PMC7162830 32162092

[jkag254-B109] Teichert I, Wolff G, Kück U, Nowrousian M. 2012. Combining laser microdissection and RNA-Seq to chart the transcriptional landscape of fungal development. BMC Genomics. 13:511. 10.1186/1471-2164-13-511. PMC3472292 23016559

[jkag254-B110] Todd JNA, Carreón-Anguiano KG, Islas-Flores I, Canto-Canché B. 2022. Fungal effectoromics: a world in constant evolution. Int J Mol Sci. 23:13433. 10.3390/ijms232113433. PMC9656242 36362218

[jkag254-B111] Torres MF et al 2016. A *Colletotrichum graminicola* mutant deficient in the establishment of biotrophy reveals early transcriptional events in the maize anthracnose disease interaction. BMC Genomics. 17:202. 10.1186/s12864-016-2546-0. PMC4782317 26956617

[jkag254-B112] Turrà D, Segorbe D, Di Pietro A. 2014. Protein kinases in plant-pathogenic fungi: conserved regulators of infection. Annu Rev Phytopathol. 52:267–288. 10.1146/annurev-phyto-102313-050143. 25090477

[jkag254-B113] Vaser R, Sović I, Nagarajan N, Šikić M. 2017. Fast and accurate de novo genome assembly from long uncorrected reads. Genome Res. 27:737–746. 10.1101/gr.214270.116. PMC5411768 28100585

[jkag254-B114] Vasselli JG et al 2022. The conidial coin toss: a polarized conidial adhesive in *Colletotrichum graminicola*. Fungal Genet Biol. 163:103747. 10.1016/j.fgb.2022.103747. 36309094

[jkag254-B115] Verma RK, Prabh ND, Sankararamakrishnan R. 2014. New subfamilies of major intrinsic proteins in fungi suggest novel transport properties in fungal channels: implications for the host-fungal interactions. BMC Evol Biol. 14:173. 10.1186/s12862-014-0173-4. PMC4236510 25112373

[jkag254-B116] Walker BJ et al 2014. Pilon: an integrated tool for comprehensive microbial variant detection and genome assembly improvement. PLoS One. 9:e112963. 10.1371/journal.pone.0112963. PMC4237348 25409509

[jkag254-B117] Wang F et al 2021. Transcription in fungal conidia before dormancy produces phenotypically variable conidia that maximize survival in different environments. Nat Microbiol. 6:1066–1081. 10.1038/s41564-021-00922-y. 34183813

[jkag254-B118] Wang X et al 2024. Zinc metalloprotease FgM35, which targets the wheat zinc-binding protein TaZnBP, contributes to the virulence of *Fusarium graminearum*. Stress Biol. 4:45. 10.1007/s44154-024-00171-z. PMC11522218 39472326

[jkag254-B119] Wardman JF, Withers SG. 2024. Carbohydrate-active enzyme (CAZyme) discovery and engineering via (ultra) high-throughput screening. RSC Chem Biol. 5:595–616. 10.1039/D4CB00024B. PMC11221537 38966674

[jkag254-B120] Werner S, Sugui JA, Steinberg G, Deising HB. 2007. A chitin synthase with a myosin-like motor domain is essential for hyphal growth, appressorium differentiation, and pathogenicity of the maize anthracnose fungus *Colletotrichum graminicola*. Mol Plant Microbe Interact. 20:1555–1567. 10.1094/MPMI-20-12-1555. 17990963

[jkag254-B121] Wight WD, Kim KH, Lawrence CB, Walton JD. 2009. Biosynthesis and role in virulence of the histone deacetylase inhibitor depudecin from *Alternaria brassicicola*. Mol Plant Microbe Interact. 22:1258–1267. 10.1094/MPMI-22-10-1258. 19737099

[jkag254-B122] Wong Sak Hoi J, Dumas B. 2010. Ste12 and Ste12-like proteins, fungal transcription factors regulating development and pathogenicity. Eukaryot Cell. 9:480–485. 10.1128/ec.00333-09. PMC2863410 20139240

[jkag254-B123] Yang Y et al 2018. A *Verticillium dahliae* pectate lyase induces plant immune responses and contributes to virulence. Front Plant Sci. 9:1271. 10.3389/fpls.2018.01271. PMC6146025 30271415

[jkag254-B124] Zhang H et al 2014. Germination and infectivity of microconidia in the rice blast fungus *Magnaporthe oryzae*. Nat Commun. 5:4518. 10.1038/ncomms5518. PMC4143928 25082370

[jkag254-B125] Zheng J et al 2023. dbCAN3: automated carbohydrate-active enzyme and substrate annotation. Nucleic Acids Res. 51:W115–W121. 10.1093/nar/gkad328. PMC10320055 37125649

