# Supplementary material for: Spore type-specific gene expression profiles underlying development and leaf infection processes of Colletotrichum graminicola
Source: G3 (Bethesda). 2026 Sep 9;16(11):jkag254. doi: 10.1093/g3journal/jkag254 (PMC13732438; doi:10.1093/g3journal/jkag254)
Supplement: Supplementary file 1 [file jkag254_supplementary_data.docx]

Supplementary Material

Spore type-specific gene expression profiles underlying development and leaf infection processes of *Colletotrichum graminicola*

Authors:

Rathi, Disha^1^; Andresen, Karsten^2^; Daniel, Rolf^3^; Guerreiro, Marco Alexandre^4,5^; Kretschmer, Matthias^6^; Kronstad, James^6^; Nowrousian, Minou^7^; Pöggeler, Stefanie^1^; Poehlein, Anja^3^; Voll, Lars M^8,9^; Nordzieke, Daniela Elisabeth^1,10^*

Affiliations:

^1^Institute of Microbiology and Genetics, Genetics of Eukaryotic Microorganisms, University of Göttingen, GZMB, Göttingen, Germany

^2^Institute of Molecular Physiology, Johannes Gutenberg University, Mainz, Germany

^3^Genomic and Applied Microbiology & Göttingen Genomics Laboratory, Institute of Microbiology and Genetics, Georg-August University of Göttingen, Göttingen, Germany

^4^Environmental Genomics Group, Botanical Institute, Christian-Albrechts University of Kiel, Kiel, Germany

^5^Max Planck Institute for Evolutionary Biology, Plön, Germany

^6^The Michael Smith Laboratories, Department of Microbiology and Immunology, University of British Columbia, Vancouver, British Columbia, Canada, V6T 1Z4

^7^Department of Molecular and Cellular Botany, Ruhr-Universität Bochum, Bochum, Germany

^8^Marburg University, Department Biology, Molecular Plant Physiology, Karl-von-Frisch-Strasse 8, D-35043 Marburg, Germany

^9^Center for Synthetic Microbiology (SYNMIKRO), Marburg University, Karl-von-Frisch-Strasse 14, D-35043 Marburg, Germany

^10^ Department of Applied Genetics and Cell Biology, Institute of Microbial Genetics,

BOKU University, Vienna, Tulln/Donau, Austria

*Corresponding author*: Daniela Elisabeth Nordzieke, [daniela.nordzieke@boku.ac.at](mailto:daniela.nordzieke@boku.ac.at)

Supplementary Figures:

Figure S1: Overview of RNA samples prepared. From each sample, including *C. graminicola* mycelium (a), falcate conidia (b), and oval conidia (c), 3 replicates were prepared. (b, c) Conidia samples were taken after different incubation timepoints including freshly harvested conidia, conidia incubated for the indicated timepoints under nutrient limiting conditions (23°C), and early maize leaf infection stages (1 dpi).

Alt text: Sample overview, with subfigures labelled from a to c, displaying the different samples used for the transcriptome analysis.

Figure S2: Heatmap displaying the expression profiles of the 100 most variable genes in freshly harvested falcate and oval conidia. Red indicates up-regulation and blue indicates down-regulation of gene expression. The top 100 variable genes were identified using iDEP.96 (Ge et al. 2018).

Alt text: Heatmap showing the expression profiles of the most regulated genes (Top 100) in falcate and oval conidia after harvest, with color codes from red (up-regulated genes) to blue (down-regulated genes).

Figure S3: 100 most variable genes in falcate and oval conidia following 5 h of incubation at nutrient limiting conditions depicted in a heatmap. Expression levels are color-coded, with red representing up-regulated and blue representing down-regulated genes. The top 100 variable genes were identified using iDEP.96 (Ge et al. 2018).

Alt text: Heatmap showing the expression profiles of the most regulated genes (Top 100) in falcate and oval conidia after 5 h of cultivation in nutrient-limiting conditions, with color codes from red (up-regulated genes) to blue (down-regulated genes).

Figure S4: Heatmap displaying 100 most variable genes in falcate and oval conidia following 16 h of incubation at nutrient limiting conditions. Up-regulated genes are depicted in red and down-regulated in blue. The top 100 variable genes were identified using iDEP.96 (Ge et al. 2018).

Alt text: Heatmap showing the expression profiles of the most regulated genes (Top 100) in falcate and oval conidia after 16 h of cultivation in nutrient-limiting conditions, with color codes from red (up-regulated genes) to blue (down-regulated genes).

Figure S5: Heatmap displaying 100 most variable genes in falcate and oval conidia harvested from maize leaves after 1 d of inoculation. The heatmap uses red and blue to denote up-regulation and down-regulation, respectively. The top 100 variable genes were identified using iDEP.96 (Ge et al. 2018).

Alt text: Heatmap showing the expression profiles of the most regulated genes (Top 100) in falcate and oval conidia after 1 d of leaf inoculation, with color codes from red (up-regulated genes) to blue (down-regulated genes).

Figure S6: Expression analysis of selected differentially expressed genes with qRT-PCR. RNA was taken from the same samples which were analyzed in the RNA-seq analysis. After DNA digestion, reverse transcription was used to generate cDNA, serving as template for the qRT-PCR reaction. Correct performance of the used oligonucleotides was tested beforehand. Base mean of the corresponding genes were: 388.88 (COGRA5_13220), 1152.99 (COGRA5_00514), 121.67 (COGRA5_09579), 260.96 (COGRA5_06545), 371.98 (COGRA5_00442), 227.90 (COGRA5_11040), 145.46 (COGRA5_14555).

Alt text: Bar chart showing qRT‑PCR expression levels for selected differentially expressed genes.

Figure S7: Differentially expressed transcription factor-encoding genes showing increased expression in oval conidia relative to falcate conidia compared to the corresponding refence conditions across different developmental stages. As reference conditions for freshly harvested conidia (FcControl, OcControl) served mycelium, for all developmental and pathogenicity-related conditions (Fc5h, Fc16h, Fc1dLF, Oc5h, Oc16h, Oc1dLF) the corresponding freshly harvested conidia samples were chosen. The top 100 variable genes were identified using iDEP.96 (Ge et al. 2018). Different color bars represent different transcription factor genes.

Alt text: Bar chart showing differentially expressed transcription factor-encoding genes with higher expression in oval conidia than in falcate conidia.

Figure S8: Heatmap to illustrate the 100 most variable expressed effector genes at different developmental stages in two conidia types. Heatmap was created using the iDEP.96 (Ge et al. 2018). Red color depicts the up-regulated genes, whereas blue represents the down-regulated genes.

Alt text: Heatmap showing the expression profiles of the most regulated effector-encoding genes (Top 100) in falcate and oval conidia, with color codes from red (up-regulated genes) to blue (down-regulated genes).

Figure S9: Regulation of effector-encoding genes during early leaf infection. The volcano plots were generated based on the comparison of gene expression data of falcate conidia leaf infection vs freshly harvested falcate conidia (a) and oval conidia leaf infection vs freshly harvested oval conidia (b). VolcaNoseR settings were as followed: fold change threshold: -2 to 2; significant threshold: 2; use thresholds to annotate: changed and significant; criterion for ranking hits: Manhattan distance. Significantly regulated are indicated in red (up-regulated) and blue (down-regulated). Locus tag numbers of the 10 most relevant hits are indicated.

Alt text: Volcano plots showing regulation of effector‑encoding genes during early maize leaf infection in oval and falcate conidia, with subfigures labelled from a to b, illustrating expression changes during early leaf infection with freshly harvested conidia as reference samples.

Figure S10: Heatmap to illustrate the 100 most variable transporter-encoding genes at different developmental stages in two conidia types. Heatmap was created using the iDEP.96 (Ge et al. 2018). Red color depicts the up-regulated genes, whereas blue represents the down-regulated genes.

Alt text: Heatmap showing the expression profiles of the most regulated transporter-encoding genes (Top 100) in falcate and oval conidia, with color codes from red (up-regulated genes) to blue (down-regulated genes).

Figure S11: Heatmap to illustrate the 100 most variable genes encoding for Carbohydrate-Active Enzymes (CAZymes) at different developmental stages in two conidia types. Heatmap was created using the iDEP.96 (Ge et al. 2018). Red color depicts the up-regulated genes, whereas blue represents the down-regulated genes.

Alt text: Heatmap showing the expression profiles of the most regulated CAZyme-encoding genes (Top 100) in falcate and oval conidia, with color codes from red (up-regulated genes) to blue (down-regulated genes).

Figure S12: Most variable differentially expressed Carbohydrate-Active Enzymes (CAZymes) in C. graminicola asexual spore types during leaf infection. Expression dynamics (log₂ fold change) of major CAZyme classes across different developmental stages and early maize leaf infection (1 dpi). As reference conditions served freshly harvested oval and falcate conidia for all time points tested for the corresponding spore type. Statistical significance was tested using a Wilcoxon test, p-values were adjusted with the Holm method. Significances displayed give differences between conidial types within each CAZyme class at the corresponding developmental stage, n = numbers of genes included. Identification of the top 100 variable genes was performed using iDEP.96 (Ge et al. 2018) based on differentially expressed genes identified (DEseq).

Alt text: Bar plot showing expression dynamics of selected CAZyme classes in falcate and oval asexual spores across developmental stages and early maize leaf infection (1 dpi).

Figure S13: Illustration of the detailed expression dynamics (log₂ fold change) of major CAZyme classes, along with their constituent families, across different developmental stages in falcate and oval conidia. As reference conditions served freshly harvested oval and falcate conidia for all time points tested for the corresponding spore type. Statistical significance was tested using a Wilcoxon test, p-values were adjusted with the Holm method. Significances provided denote differences

between conidial types. The statistical significance indicates the differences between conidial types within each CAZyme class at the respective developmental stage.

Alt text: Box Plot showing detailed expression dynamics of major CAZyme classes and their families in falcate and oval conidia across developmental stages.

Figure S14: Heatmap displaying gene expression of selected developmental genes in oval and falcate conidia after different developmental timepoints and early leaf infection. Differential expression was determined with a log2 fold change ≤ –1 or ≥ 1 and an adjusted p-value (padj) < 0.05. The heatmap was generated using iDEP.96, with red indicating up-regulated genes and blue indicating down-regulated genes.

Alt text: Heatmap showing the expression profiles of selected genes relevant for fungal development in falcate and oval conidia, with color codes from red (up-regulated genes) to blue (down-regulated genes).

Figure S15: Heatmap highlighting known secondary metabolite gene clusters associated with the biosynthesis of fusaridione A, depudecin, and phomopsins (A, B, E), which are induced during leaf infection (1dpi) in both C. graminicola spore types. Red color depicts the up-regulated genes, whereas blue represents the down-regulated genes. The heatmap was generated using iDEP.96, with red indicating up-regulated genes and blue indicating down-regulated genes.

Alt text: Heatmap showing the expression profiles of selected secondary metabolite biosynthesis clusters in falcate and oval conidia, with color codes from red (up-regulated genes) to blue (down-regulated genes).

Supplementary Tables

Table S1: Description and mapping rates of RNA-seq samples. Wt in this table refers to CgM2 wild type like strain of *Colletotrichum graminicola* (M1.001). Reads were mapped to the *C. graminicola* genome version V5 with the mapping rate (in %) given in the last column. Mapping rates range from 93 to 98 % except for samples 11-16 (LF and LO), where mapping rates are much lower than for the other samples. The reason for the low mapping rates in samples 11-16 is because these samples are derived from maize plants infected with falcate or oval conidia, and the majority of reads from these samples map to the plant genome (Mapping to confirm this was done to the genome of *Zea mays*, acc. no. GCF_902167145.1, to which 89-91 % of reads from samples 11-16 mapped).

| **Sample#** | **Sample description and abbreviation for downstream analysis** | **Number of reads** | **Mapping rate in %** |
| --- | --- | --- | --- |
| 2 | oval conidia after incubation for 5 h under nutrient limiting conditions (**O5**) | 24008487 | 97.7 |
| 3 |  | 19847895 | 98.1 |
| 4 |  | 20828345 | 97.7 |
| 5 | mycelium (**M**) | 20222305 | 97.7 |
| 6 |  | 28996415 | 96.2 |
| 7 |  | 22877299 | 97.0 |
| 8 | freshly harvested oval conidia (**O**) | 21225957 | 97.1 |
| 9 |  | 27769533 | 97.2 |
| 10 |  | 27985395 | 97.4 |
| 11 | leaf infection with falcate conidia 1dpi (**LF**) | 222299954 | 0.46 |
| 12 |  | 178061742 | 0.94 |
| 13 |  | 186226237 | 2.87 |
| 14 | leaf infection with oval conidia 1dpi (**LO**) | 207168903 | 0.74 |
| 15 |  | 189665886 | 0.81 |
| 16 |  | 158565123 | 0.69 |
| 17 | freshly harvested falcate conidia (**F**) | 21474610 | 94.6 |
| 18 |  | 19909295 | 93.6 |
| 19 |  | 24700766 | 94.6 |
| 20 | falcate conidia after incubation for 5 h under nutrient limiting conditions (**F5**) | 25167917 | 98.1 |
| 21 |  | 24226438 | 97.8 |
| 22 |  | 21012590 | 97.7 |
| 23 | falcate conidia after incubation for 16 h under nutrient limiting conditions (**F16**) | 22586828 | 98.1 |
| 24 |  | 22042925 | 97.8 |
| 25 |  | 26584963 | 97.6 |
| 26 | oval conidia after incubation for 16 h under nutrient limiting conditions (**O16**) | 26694901 | 98.3 |
| 27 |  | 21464125 | 95.8 |
| 28 |  | 26458053 | 94.7 |

Table S2: Pathway enrichment analysis performed for freshly harvested conidia and early maize leaf infection. Pathway enrichment analysis was performed using the GAGE method implemented in iDEP.96 (log2 fold change <=-1 or >=1, padj <0.05). Gene sets ranging from 5 to 2,000 genes were included. Pathways were considered significant at an FDR cutoff of 0.05. Prior to enrichment analysis, genes with FDR ≥ 1 were excluded. Pathways with blue labelled up are up-regulated, whereas pathways with red labelled down are down-regulated in specified samples.

| **Pathway** | **Fc control** | **Fc LF** | **Oc Control** | **Oc LF** |
| --- | --- | --- | --- | --- |
| Secondary metabolic process | Down | Up | Down | Up |
| Mycotoxin biosynthetic process | Down | Up | Down | Up |
| Hydrolase activity, acting on glycosyl bonds | Down | Up | Down | Up |
| Carbohydrate metabolic process | Down | Up | Down | Up |
| Oxidoreductase activity | Down | Up | Down | Up |
| rRNA processing | Up | Down | Up | Down |
| Cellular response to stress | Up | Down | Up | Down |
| Cellular response to DNA damage stimulus | Up | Down | Up | Down |
| Nucleolus pathways | Up | Down | Up | Down |
| Protein dimerisation activity | Up | Down | Up | Down |

Table S3: Oligonucleotides used in this study.

| **Oligonucleotide** | **Sequence (5‘ to 3‘)** |
| --- | --- |
| gapdh_fw  gapdh_rv  COGRA5_06545_fw  COGRA5_06545_rv  COGRA5_14555_fw  COGRA5_14555_rv  COGRA5_11040_fw  COGRA5_11040_rv  COGRA5_00514_fw  COGRA5_00514_rv  COGRA5_00442_fw  COGRA5_00442_rv  COGRA5_09579_fw  COGRA5_09579_rv  COGRA5_13220_fw  COGRA5_13220_rv | CCACCGACATGATCGGCAAC  GGGAGTAACCCCACTCGTTG  TGACACCTCCAACCAGCTTA  GTCCCAATCATCGAGGCAAG  GTCTACAACGAGCTGGCAAG  CTCTCCCGAACCTAACCCAG  CTGCCGACCTGAAATGGATG  CCCAAACGTACCTCCAAACC  GGGGTTCAACGGCAAGACTG  TGTCCTCGCACCAAGAATCTG  CTTACCAACTTCGCTTTGACAGG  GCCACCAATCGCATCGTTC  ACGAGACGTTGTGGGACGT  GAACCAGAGCGCTCTCGATC  CGACGTCTTGTACTTCGCGAC  GCAGCTCTTGAGGATGAGGC |

Table S4: Effector genes showing the highest spore-type dependent specificity during early leaf interactions of *C. graminicola*. Evaluation based on the Top 100 regulated effector genes (Figure H File S1). Genes were included when the log2 fold change was >2 comparing early leaf infection samples of falcate with early leaf infection samples of oval conidia (LF_vs_LO) and padj was <0.05. Reference conditions as indicated.

| **locus_tag** | **base mean** | **LO_vs_O log2FoldChange** | **LF_vs_F log2FoldChange** | **LF_vs_LO log2FoldChange** | **effector type** | **reference** |
| --- | --- | --- | --- | --- | --- | --- |
| COGRA5_06182 | 274.19 | -13.14809737 | 1.604954176 | 22.6848376 | Apoplastic effector | - |
| COGRA5_10559 | 172.60 | -7.162916069 | -2.210787695 | 6.992890832 | Apoplastic effector | (Kleemann et al. 2012, Oome and Van den Ackerveken 2014, Torres et al. 2016, Seidl and Van den Ackerveken 2019) |
| COGRA5_04185 | 23037.02 | -0.737109579 | 6.636901253 | 3.193825201 | Apoplastic effector | (Bettini et al. 2012, Eisermann et al. 2019) |

References

**Bettini PP et al.** Identification and characterization of GEO1, a new class II hydrophobin from *Geosmithia* spp. Canadian journal of microbiology. 2012; 58(8): 965-972. <https://doi.org/10.1139/w2012-069>

**Eisermann I et al.** Two genes in a pathogenicity gene cluster encoding secreted proteins are required for appressorial penetration and infection of the maize anthracnose fungus *Colletotrichum graminicola*. Environmental microbiology. 2019; 21(12): 4773-4791. <https://doi.org/10.1111/1462-2920.14819>

**Kleemann J et al.** Sequential delivery of host-induced virulence effectors by appressoria and intracellular hyphae of the phytopathogen *Colletotrichum higginsianum*. PLoS pathogens. 2012; 8(4): e1002643. <https://doi.org/10.1371/journal.ppat.1002643>

**Oome S and Van den Ackerveken G.** Comparative and functional analysis of the widely occurring family of Nep1-like proteins. Molecular plant-microbe interactions. 2014; 27(10): 1081-1094. <https://doi.org/10.1094/MPMI-04-14-0118-R>

**Seidl MF and Van den Ackerveken G.** Activity and phylogenetics of the broadly occurring family of microbial Nep1-like proteins. Annual review of phytopathology. 2019; 57(1): 367-386. <https://doi.org/10.1146/annurev-phyto-082718-100054>

**Torres MF et al.** A *Colletotrichum graminicola* mutant deficient in the establishment of biotrophy reveals early transcriptional events in the maize anthracnose disease interaction. BMC genomics. 2016; 17(1): 202. <https://doi.org/10.1186/s12864-016-2546-0>
